# Diversification in the inositol tris/tetrakisphosphate kinase (ITPK) family: crystal structure and enzymology of the outlier *At*ITPK4

**DOI:** 10.1042/BCJ20220579

**Published:** 2023-03-10

**Authors:** Hayley L. Whitfield, Sining He, Yinghong Gu, Colleen Sprigg, Hui-Fen Kuo, Tzyy-Jen Chiou, Andrew M. Riley, Barry V.L. Potter, Andrew M. Hemmings, Charles A. Brearley

**Affiliations:** 1 School of Biological Sciences, University of East Anglia, Norwich Research Park, Norwich NR4 7TJ, U.K; 2 Department of Biology, School of Life Sciences, Southern University of Science and Technology, Nanshan, Shenzhen 518055, China; 3 Agricultural Biotechnology Research Center, Academia Sinica, Taipei 115, Taiwan; 4 Medicinal Chemistry & Drug Discovery, Department of Pharmacology, University of Oxford, Mansfield Road, Oxford OX1 3QT, UK; 5 College of Food Science and Technology, Shanghai Ocean University, Shanghai 201306, China

**Keywords:** inositol polyphosphates, ATP Grasp, HAD domain, X-ray crystallography, Enzymology, Molecular Interactions, Structural Biology

## Abstract

*Myo*-inositol tris/tetrakisphosphate kinases (ITPKs) catalyze diverse phosphotransfer reactions with *myo*-inositol phosphate and *myo*-inositol pyrophosphate substrates. However, the lack of structures of nucleotide-coordinated plant ITPKs thwarts a rational understanding of phosphotransfer reactions of the family. Arabidopsis possesses a family of four ITPKs of which two isoforms, ITPK1 and ITPK4, control inositol hexakisphosphate and inositol pyrophosphate levels directly or by provision of precursors. Here, we describe the specificity of Arabidopsis ITPK4 to pairs of enantiomers of diverse inositol polyphosphates and show how substrate specificity differs from Arabidopsis ITPK1. Moreover, we provide a description of the crystal structure of ATP-coordinated AtITPK4 at 2.11 Å resolution that along with description of the enantiospecificity of the enzyme affords a molecular explanation for the diverse phosphotransferase activity of this enzyme. That Arabidopsis ITPK4 has a *K*_m_ for ATP in the tens of micromolar range, potentially explains how, despite the large-scale abolition of InsP_6_, InsP_7_ and InsP_8_ synthesis in *Atitpk4* mutants, *Atitpk4* lacks the phosphate starvation responses of *Atitpk1* mutants. We further demonstrate that Arabidopsis ITPK4 and its homologs in other plants possess an N-terminal haloacid dehalogenase-like fold not previously described. The structural and enzymological information revealed will guide elucidation of ITPK4 function in diverse physiological contexts, including InsP_8_-dependent aspects of plant biology.

## Introduction

*Myo*-inositol pyrophosphates (diphosphoinositol phosphates) are present in plants at levels that are a small fraction of their precursor, *myo*-inositol hexakisphosphate InsP_6_. Like inositol phosphates and nucleotides, they are responsive to phosphate resupply after chronic phosphate starvation [1]. The catalytic activities of inositol hydroxy- and phosphate kinases, IPMK, ITPK1, IPK1 and VIH1/2 (also known as VIP1/2) are responsible for InsP_6_ and inositol pyrophosphate synthesis in Arabidopsis and contribute to the control of phosphate homeostasis [2–10] and the phosphate starvation response (PSR) whereby the Myb transcription factor PHR1 controls the expression of a host of genes regulating plant response to phosphate supply [6,11] (Figure S1).

While early reports suggested that Pi is a ligand of the protein SPX1 whose interaction with PHR1 sequesters the latter from its activating interaction with the P1BS element of PSR genes [12], InsP_6_ and inositol pyrophosphates are much tighter binding ligands of SPX1 [13]. In vitro studies of SPX1 orthologs show remarkably little discrimination in strength of binding between InsP_6_ and inositol pyrophosphates [13], and some of the outputs proposed of inositol pyrophosphate interaction with SPX1 are met by InsP_6_ [4,10,14]. Nonetheless, a current model of PSR attributes special function to InsP_8_ [14]. A metabolic perspective is, nevertheless, provided by the global inositol phosphate response to resupply of phosphate after chronic starvation [1]. Here, the amplified response of InsP_8_, relative to InsP_7_ and InsP_6_, points to metabolic flux from these precursors and possibly also from InsP_3_, InsP_4_ and InsP_5_ species that are also increased on Pi resupply. That inositol pyrophosphate synthesis is coupled to the metabolism of lower inositol phosphates was proposed [9].

Even so, studies suggest that InsP_8_ or the activity of the enzymes that make it do not necessarily correlate with PSR. Under Pi-limiting conditions *Vip1-2/vip2-2* seedlings lack PSR [15], showing quite different behavior to reported *vih* mutants [4] [10] [14]. Kuo and coworkers also did not observe constitutive PSR in Pi-replete *vih1-1*, *vih2-3*, *vih2-4*, *vip1-1*, *vip1-2*, *vip2-1*, *vip2-2*, *vip1-2/vip2-1* mutants [6]. Here, the different alleles take different names depending on their source (Table S1 [6]). Moreover, *itpk4* mutants, which show pronounced reductions in InsP_6_ [6,16] are severely depleted in InsP_7_ and InsP_8_ [6] and lack the constitutive PSR established for *itpk1* and *ipk1* mutants under Pi-replete conditions [17]. These differences may arise from the pleiotropic consequences of disruption of inositol phosphate, including InsP_6_ and inositol pyrophosphate, synthesis [18]. Inositol phosphates are participants in biotic and abiotic interactions of plants [18–21]. The effects of mutation of Itpk1 and Ipk1 extend to influence on plant immunity mediated by salicylic acid [19,20], a property shared by Vih2 mutants [19] and other mutants of InsP_6_ synthesis [20]. Itpk1 mutants also show auxin-related phenotypes that may relate to combinatorial effect of altered inositol phosphate and inositol pyrophosphate species in these plants [21].

Here, we combine structural biology, enzyme assay and LC-ICP-MS to characterize AtITPK4 and *Atitpk4* mutants. We reveal novel facets of ITPK structure, substrate preference and kinetic behavior that set ITPK4 apart from other inositol hydroxy- and phosphate kinases involved in inositol pyrophosphate synthesis in plants.

## Results

### The enantiomeric discrimination of AtITPK4 towards inositol phosphates is the opposite of ITPK1: ITPK4 prefers Ins(1,4,6)P_3_

Despite a resurgence in interest in the role of inositol phosphates and pyrophosphates in plants, particularly in context of Pi homeostasis, there are remarkably few studies of the isomeric complement of potential physiological substrates of inositol hydroxyl- or inositol phosphate-kinases of plants. Nonetheless, the dominant Ins(3,4,5,6)P_4_ 1-kinase activity of Arabidopsis ITPK1 [9] fits well with historic analysis of inositol phosphate stereoisomerism and enantiomerism [22–24]. Previous characterization of the ITPK family highlighted that within Arabidopsis ITPK4 is an outlier to the family [25], and more broadly that ITPK’s have diverse phosphotransfer capabilities [26–29]. The latter observation is important since phosphotransfer (from inositol phosphate or pyrophosphate) to water defines phosphatase activity, while transfer to ADP is a facet of reversibility exemplified by IPK1 and ITPK1 [1,9,30,31]. The ITPK family additionally shows ‘phosphoisomerase’ or ‘phosphomutase’ activity [25,26,29]. We therefore tested Arabidopsis ITPK4 for hydroxy-kinase and phosphate-kinase activity and for other phosphotransferase activities.

**Figure 1:**
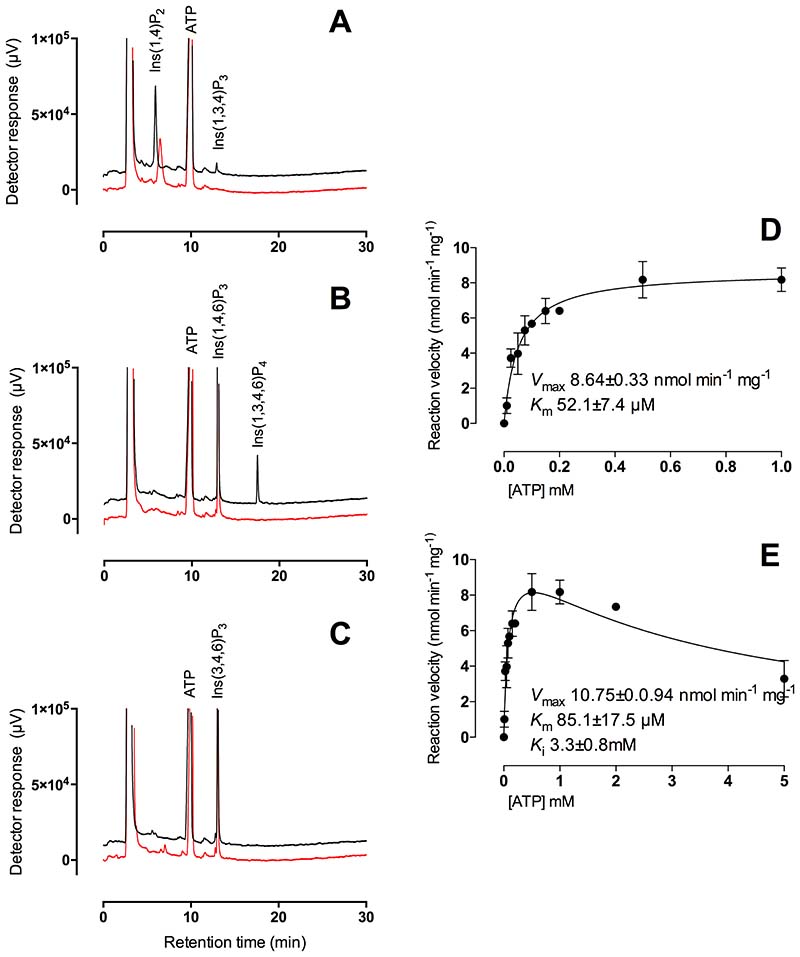
Hydroxy-kinase reactions catalyzed by *At*ITPK4. (**A**) Ins(1,4)P_2_, (**B**) Ins(1,4,6)P_3_ and (**C**) Ins(3,4,6)P_3_ incubated with (black line) or without protein (red line). Products of 20 min reactions were analyzed by HPLC. These assays were set up to distinguish the use of different substrates. (**D**), (**E**) Michaelis-Menten kinetic parameters for hydroxy-kinase action of AtITPK4 on Ins(1,4,6)P_3_. Reaction conditions were set to limit substrate consumption to less than 10%. Analysis of the kinase activity of AtITPK4 to these substrates has been performed on at least five separate occasions by these methods.

The substrate preference of Arabidopsis ITPK4 towards a variety of inositol phosphate substrates and the identities of products generated is shown (Table 1). The structures of these molecules are shown (Figure S2). ITPK4 has preferential activity against ‘lower’ inositol phosphates (Figure 1, Figure S3). Like others [1] we found no evidence of inositol phosphate-kinase (pyrophosphorylating) activity (see below). Activity against Ins(1,4)P_2_, Ins(1,4,6)P_3_ and Ins(3,4,6)P_3_ was tested with an ATP regenerating assay revealing product conversion activities of 6.8%, 10.4% and 0%, respectively (Figure 1). Consequently, the preference of ITPK4 for Ins(1,4,6)P_3_ over Ins(3,4,6)P_3_ is opposite to that of Arabidopsis ITPK1 [9] and we offer a structural explanation of this below. Nonetheless, extended incubation revealed ITPK4 could use Ins(3,4,6)P_3_ as a weak substrate (Table 1). The products of phosphorylation of Ins(1,4)P_2,_ Ins(1,4,6)P_3_ and Ins(3,4,6)P_3_ were identified as Ins(1,3,4)P_3_, Ins(1,3,4,6)P_4_ and Ins(1,3,4,6)P_4_, respectively, by spiking with standards and by comparison of previously reported separations of inositol phosphates [9,32]. The enantiomeric character of individual pairs of substrates is shown (Figure S2). Early characterization of enzymes of this class in plants considered them as Ins(1,3,4)P_3_ 5/6 kinases [25–29], here Ins(1,3,4)P_3_ is a weak substrate for AtITPK4 when compared with Ins(1,4,6)P_3_ (Figure S3).

### Substrate inhibition of *At*ITPK4 by ATP

Kinetic assays of ITPK4 against Ins(1,4,6)P_3_, limited to less than 10% substrate turnover, revealed a *K*_m_ for ATP of 52 ± 7 µM and *V*_max_ 8.64 ± 0.33 nmol min^-1^ mg^-1^ when fitted to the Michaelis-Menten equation (Figure 1D). This reveals a striking difference between Arabidopsis ITPK1 and Arabidopsis ITPK4 for their preferred substrates. The latter has a 15 fold lower *K*_m_ ATP and a 1000 fold lower *V*_max_. ITPK1 prefers Ins(3,4,5,6)P_4_, and displays *K*_m_ ATP of 1.22 mM, V_max_ 8640nmol min^-1^ mg^-1^ [9]. Riemer et al [1] reported kinetic parameters for AtITPK1 of *K*_m_ ATP with InsP_6_ of 0.52 mM and *V*_max_ 18 nmol min^-1^ mg^-1^. Thus, Arabidopsis ITPK4 activity towards Ins(1,4,6)P_3_ is comparable to Arabidopsis ITPK1 activity towards InsP_6_ and this is several orders of magnitude less than ITPK1 activity towards Ins(3,4,5,6)P_4_. A recent report afforded *K*_m_ InsP_6_ of 0.025 mM and *K*_m_ ATP of 0.21-36 mM, with *V*_max_ in the low tens of nmol min^-1^ mg^-1^, for *Zea mays* inositol tris/tetrakisphosphate kinase 1, *Zm*ITPK1 [33]. Activity against ‘lower’ inositol phosphates was not described.

As well as a low *K*_m_ ATP, Arabidopsis ITPK4 showed inhibition at high ATP concentrations where data fitted to a substrate inhibition model in GraphPad gave a *K*_i_ of 3.3 mM (Figure 1E). Such inhibition was not seen for AtITPK1, tested up to 10mM ATP [9], though inhibition is seen at 10 mM in the data of Riemer et al. [1]. It is tempting to speculate that the difference in *K*_m_ ATP for ITPK1 and ITPK4 underlies the difference in PSR of the respective mutants, since both are essential for inositol pyrophosphate synthesis either directly from InsP_6_ (ITPK1 [1,9]) or from their contribution to InsP_6_ synthesis (ITPK1 and ITPK4 [1,6,9,16].

### AtITPK4 does not phosphorylate IP_5_ or IP_6_

We tested a range of substrates under ATP-regenerating and non-regenerating assay conditions. Unlike Arabidopsis ITPK1, which converts Ins(1,2,3,4,5)P_5_ to putative 5PP-Ins(1,2,3,4)P_4_ [9], Arabidopsis ITPK4 did not phosphorylate IP_6_ (Figure S4) nor did it phosphorylate any of the six InsP_5_ isomers (Table 1). The putative 5PP-Ins(1,2,3,4)P_4_ product (of *At*ITPK1) may be the novel unidentified PP-InsP_4_ detected in Arabidopsis [1]. *Zm*ITPK1, however, converts Ins(1,2,3,4,5)P_5_ to 3PP-Ins(1,2,4,5)P_4_ with ca. 25% of the activity towards InsP_6_. It also displays apparent pyrophosphorylating activity against multiple InsP_5_ isomers in a coupled phosphate-releasing assay [33]. Interestingly, 3PP-Ins(1,2,4,5)P_4_ elutes just before 5-InsP_7_, which elutes after InsP_6_ on Partisphere SAX HPLC (see Figures 1,2 of [33]), while the putative 5PP-Ins(1,2,3,4)P_4_ elutes before InsP_6_ on the same column (see Figure S7 of [9]). *Zm*ITPK1 shares less identity with the ATP Grasp fold of Arabidopsis ITPK4 than does *Hs*ITPK1. These observations highlight the importance of effective means of discriminating products, which can be achieved in a simple manner by HPLC and post-column detection with ferric nitrate [9] or HPLC and radio-detection [9,33].

Arabidopsis ITPK4 was however able to use Ins(1,4)P_2_, Ins(1,4,6)P_3_ and Ins(3,4,5)P_3_ as substrates and additionally Ins(1,3,4)P_3_, Ins(3,4,6)P_3_ and Ins(1,4,5,6)P_4_ as weak substrates. It did not use Ins1P, Ins3P, Ins(1,4,5)P_3_, Ins(1,3,4,6)P_4_ or Ins(3,4,5,6)P_4_ under non-regenerating assay conditions when incubated for 2 hours (Table 1). Again, the preference of Arabidopsis ITPK4 for enantiomers is opposite to that of Arabidopsis ITPK1, it prefers Ins(1,4,6)P_3_ over Ins(3,4,6)P_3_ and Ins(1,4,5,6)P_4_ over Ins(3,4,5,6)P_4_. We caution against assertions of activity of the ITPK family within and between species without exhaustive testing, the ITPK family is remarkable for its catalytic flexibility.

### *At*ITPK4 regulates InsP_6_, InsP_7_ and InsP_8_ synthesis

**Figure 2:**
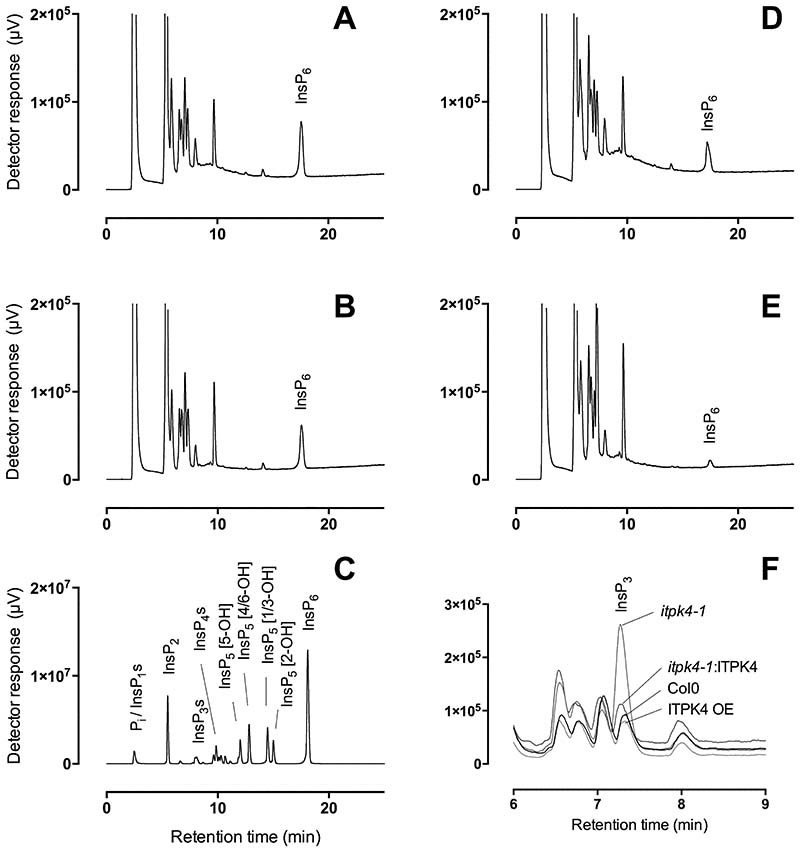
Inositol phosphates in mature vegetative tissue. Extracts of (**A**) Col0, (**B**) ITPK4-YFP206 complemented itpk4-1, (**C**), ITPK4 overexpression line, (**D**), itpk4-1, were analysed by HPLC-ICP-MS. (**E**) acid-hydrolysed InsP6 standards. (**F**) Comparison of resolved peaks in the InsP_3_ region of chromatograms (A-D). The traces shown are representative of triplicate determinations for this experiment. Individual genotypes have been analysed on at least one other occasion by this method.

*Atitpk4* mutants have been characterized in detail. They lack the phosphate over-accumulation of *Atipk1* and *Atitpk1*. They lack PSR, and they show reduced seed InsP_6_ [6] [16]. TiO_2_-PAGE analysis also showed greatly reduced vegetative tissue InsP_6_, InsP_7_ and InsP_8_ [17]. To test for effect of Itpk4 mutation on inositol phosphates, we extracted inositol phosphates in perchloric acid, concentrated on TiO_2_ and analysed by LC-ICP-MS (Figure 2). This approach retains the considerable resolving power of HPLC on acid-eluted CarboPac PA200 both for inositol phosphates and inositol pyrophosphates. It resolves 5-InsP_7_, 4/6-InsP_7_ and 1/3-InsP_7_ [9]. We did not detect InsP_7_ or InsP_8_ with column-loading of extracts equivalent to approximately 160 mg of soil-grown *itpk4-1*, ITPK4-OE line or ITPK4-YFP-complemented *itpk4-1* [6,17]. The levels of InsP_6_ in *itpk4-1* were significantly different (*P*< 0.05) from that of Col0 and ITPK4-OE, with mean and s.d. of 26 ± 1, 153 ± 39 and 181 ± 43 pmol g^-1^ f. wt., respectively. A single ITPK4-YFP-complemented *itpk4-1* gave a value of 118 pmol g^-1^ f. wt. The reduction of InsP_6_ content is consistent with [^3^H]-*myo*-inositol- and [^32^P]-P_i_-labelling of seedlings [6], with TiO_2_-PAGE analysis of mature vegetative tissues [17] and with measurements in seeds [6]. Moreover, the absolute levels in vegetative tissues match that measured by CE-MS in P_i_-deplete, hydroponically grown Col0, approximately 150 pmol g^-1^ f. wt. [1]. These data validate LC-ICP-MS for measurement of inositol phosphates in vegetative tissues, the site of PSR, without recourse to radiolabeling. They further draw attention to the lack of PSR in *itpk4* mutants [6,17].

Reduction of InsP_6_ levels in *itpk4-1* was accompanied by increases in a peak with the chromatographic properties of an InsP_3_ (Figure 2E, F). These increases were reversed by complementation with ITPK4 (Figure 2D, F and absent in the ITPK4-overexpression line (Figure 2B, F).

### The crystal structure of *At*ITPK4

The foregoing enzymological and physiological characterization of ITPK4 and *itpk4* mutants again sets ITPK4 apart from other ITPK family members. To provide a structural context for these observations, we sought crystal structures of *At*ITPK4 and *At*ITPK1. We anticipated that these structures would help facilitate understanding of the variation in substrate preference between the two. No crystal structure for an ITPK4 exists but crystal structures of wild-type ITPK1 enzymes from *Entamoeba histolytica* (*Eh*ITPK1) [34] and *Homo sapiens* (*Hs*ITPK1) [27] are available. For plant enzymes, a medium-resolution (2.9 Å) structure of the wild-type apo enzyme from maize (*Zm*ITPK1) has been reported along with a 2.6 Å resolution structure of an InsP_6_-coordinated H192A mutant [33]. These structures of *Zm*ITPK1 lack electron density for groups of residues that we show below form part of the binding pocket for nucleotide in *At*ITPK4.

We were unable to crystallize *At*ITPK1, whose enzymology is described [9], but were successful in obtaining a crystal structure of AtITPK4 in complex with ATP using the structure of *Eh*ITPK1 (PDB entry 1Z2N) as a search model in molecular replacement. The structure was solved in space group P2_1_2_1_2_1_ with a monomer of the enzyme in the asymmetric unit. Refined against all data to 1.91 Å resolution, this gave a final model with an R-factor of 21.0% (R_free_ 24.1%) (Table 2).

**Figure 3:**
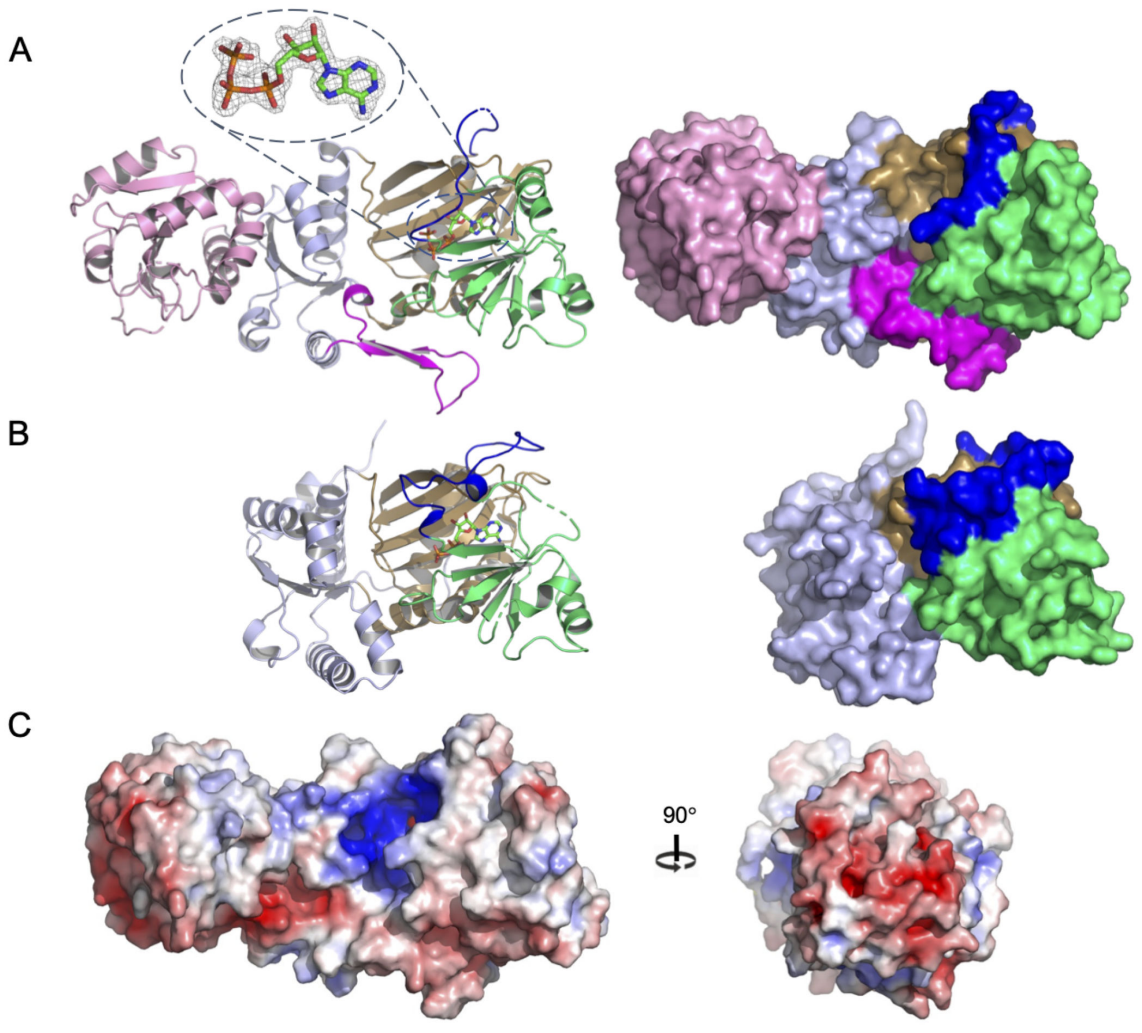
An overview of the crystal structure of *At*ITPK4. **(A)** Left panel, cartoon representation of structure of *At*ITPK4 coloured by domain: HAD domain (pink), kinase N-terminal domain (light blue), kinase central domain (lime green) and kinase C-terminal domain (sand). The residues of the tab insertion in *At*ITPK4 are coloured magenta while those of the tether are shown in blue. Broken lines in the backbone trace indicate residues unresolved in the model due to disorder. The sidechains of residues forming the unique ion pair in *At*ITPK4 are shown in stick format with interactions indicated by dotted black lines. Bound ATP is shown in stick format with atom colouring as follows: carbon-green, oxygen-red, nitrogen-blue and phosphorus-orange. Inset: the 2Fo-Fc electron density map (grey mesh) in the region of ATP is contoured at 1.5 σ. Right panel, molecular surface representation of the structure of *At*ITPK4 coloured by domain and insertion. **(B)** Left panel, cartoon representation of the structure of human ITPK1 (*Hs*ITPK1). Bound ADP is shown in stick format. The orientation of view, and atom and domain colouring follows that shown in panel (a). Right panel, molecular surface representation of the *Hs*ITPK1 structure coloured by domain and insertion. **(C)** Left panel, a view of the molecular surface of *At*ITPK4 coloured by electrostatic potential (red-acidic, blue-basic). The orientation of the molecule is the same as that in panel (A). Right panel, view of the electrostatic potential of the HAD-like domain. The view represents a 90° rotation to that shown the left panel such that the HAD-like domain is viewed head on.

The structure consists of a canonical C-terminal ATP Grasp kinase domain (residues 151-423) and an N-terminal domain (residues 1-150) which adopts a haloalkane dehalogenase (HAD)-like fold (Figure 3). This latter domain is not conserved in any other ITPK family member and its structure was built in stages by careful manual fitting to difference electron density maps interspersed with rounds of refinement. Despite this, the quality of the fit of the final refined model of this domain to difference electron density maps was generally lower than that seen in the kinase domain. The average refined atomic temperature factors for the HAD-like and kinase domains are 76.7 Å^2^ and 36.1 Å^2^, respectively, reflecting a generally higher degree of flexibility in the former. Nevertheless, the topology of the domain is clearly indicated by the corresponding electron density maps (Figure S5) and geometrical indicators are in accordance with correct chain tracing. This degree of inherent mobility localized to a domain within an otherwise ordered protein structure is not unusual e.g. ([35] and [36]) and may indicate either a degree of intrinsic disorder in the domain or the absence of a stabilizing binding partner in the crystal, be it a lower molecular weight ligand or other protein(s) that would otherwise participate as part of either a homo- or heteroprotein complex. In this respect, we found no evidence that *AtI*TPK4 engages in homo-oligomerization, a property described for other HAD superfamily proteins (e.g., [37]).

### Comparison of the kinase domains of *At*ITPK4 and *At*ITPK1 suggests a structural basis for enantiospecificity

**Figure 4:**
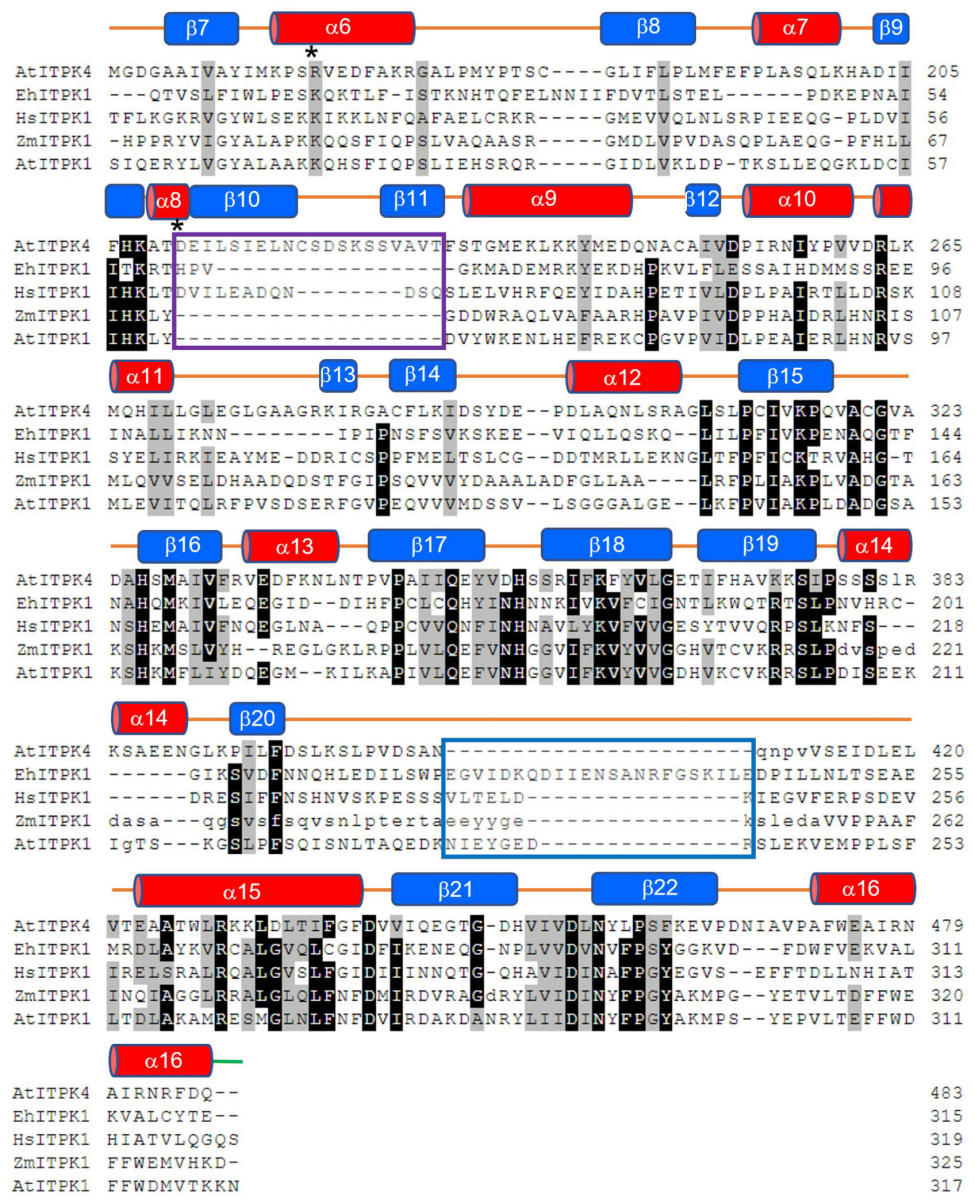
Structure-based sequence alignment of ITPKs. *At*ITPK4- *Arabidopsis thaliana* ITPK4 (PDB entry 7PUP), *Eh*ITPK1- *Entamoeba histolytica* ITPK1 (PDB entry 1Z2P), *Hs*ITPK1- *Homo sapiens* ITPK1 (PDB entry 2QB5), *Zm*ITPK1- *Zea mays* ITPK1 (PDB entry 7ZN5), *At*ITPK1- *Arabidopsis thaliana* ITPK1 (Alphafold Protein Structure Database entry Q9SBA5). Residue single letter codes shown in lower case for residues unresolved in structures of *At*ITPK4 and *Zm*ITPK1. Note that the alignment of the unresolved residues in *Zm*ITPK1 follows that predicted by the corresponding Alphafold model. Residues with at least 80% homology are shown with black backgrounds, while those conserved by residue class in at least four of the five sequences are shown with light grey backgrounds. Secondary structural elements of *At*ITPK4 are indicated by red cylinders (α-helices) or by blue boxes (β-strands) and labelled. The regions of the tab and tether insertions are enclosed in magenta and blue boxes, respectively. Residues forming the unique active site ion pair in *At*ITPK4 are indicated by asterisks (*).

The kinase domain of *AtI*TPK4 possesses the familiar ATP Grasp fold found in all previously described ITPK family members (Figure S6) and features the expected highly positively charged active site (Figure 3C) The overall structure is made up of three conserved subdomains which we shall refer to as N-terminal, central and C-terminal following the nomenclature previously applied in descriptions of the crystal structures of ITPK1 orthologs [27,33,34] (Figure 3). Comparative analysis using all-by-all flexible pairwise structure alignments between the kinase domain of *AtI*TPK4 and the available ITPK1 crystal structures revealed human ITPK1 to be the most similar (PDB entry 2QB5; RMSD 2.95 Å; 21.0 % sequence identity for 306 structurally equivalenced residues) (Figure 4). While the enzyme from *E.histolytica* had a slightly lower RMSD (PDB entry 1Z2N; 2.85 Å) it showed both a lower number of structurally equivalenced residues (280) and a lower percentage identity (15.9 %). The maize ITPK1 structure shows the lowest structural and sequence homology (PDB entry 7TN5; RMSD 3.04 Å; 264 equivalenced residues; 16.2 % identity). Unsurprisingly, the predicted structure of *At*ITPK1 taken from the Alphafold Protein Structure Database [38] also has an ATP Grasp fold. Alphafold predictions of protein structures have been shown to be of high accuracy [39]. Indeed, we note an RMSD of 0.64 Å over 287 aligned Cα atoms between Alphafold-predicted and observed structures of the kinase domain of *At*ITPK4 demonstrating the predictive power of the method. However, despite this, we have chosen not to directly compare the predicted structure of AtITPK1 with the crystal structure of *At*ITPK4 but rather to draw on it only for qualitative indications of overall fold and sequence alignment.

In terms of the overall structures of their kinase domains, *At*ITPK4 and the ITPK1 (including *At*ITPK1) have two major areas of divergence; the first is an insertion in the central subdomain of the ITPK1s relative to *At*ITPK4, and the second is an insertion in the N-terminal subdomain of *At*ITPK4 relative to ITPK1s (Figures 3,4). The insertion in the ITPK1s is found in the polypeptide connection between the C-terminal β-strand of the central domain (strand β20) and a helix (α15) of the C-terminal domain, the former domain known to adjust its position on nucleotide and substrate binding [40]. This polypeptide lies across the top of the active site cavity linking the two domains and for this reason we refer to it as the ‘tether’. In all the ITPK1s of known molecular structure where this polypeptide is resolved it provides residues which help bind and orient ATP and are positioned to interact with a bound substrate molecule. This region is disordered in the crystal structure of *Zm*ITPK1, however, sequence alignment indicates an insertion relative to AtITPK4 at this site (Figure 4). The insertion in the tether polypeptide of *At*ITPK1 is predicted to be eight amino acids in length relative to *At*ITPK4 and potentially provides residues such as Asn234, glutamates at 236 and 239, and Arg240 to line the active site cleft. The second divergent feature is a unique insertion in *At*ITPK4 which folds to form a two-stranded β-sheet and connecting polypeptide loop which we refer to as the ‘tab’ lying on the domain surface (Figure 3). This feature is well conserved in ITPK4-like proteins from Brassicaceae (Figure S7) (however, we acknowledge the lack of experimental verification of the substrate specificity of the homologues we identified). Compared to the ITPK1s, it reorganizes one end of the active site cleft and introduces a highly conserved salt bridge between Asp211 and Arg165, the latter of these residues pointing towards the catalytic centre. Both the tab and tether insertions help shape the active site cleft and contribute residues to substrate specificity pockets, suggesting they may contribute to differential substrate recognition. The net result of these differences is that AtITPK4 has an active site which is more open than that of *Eh*ITPK1, and particularly so compared to the very enclosed pocket of *Hs*ITPK1 (Figure S8). The narrow active site in the predicted structure of *At*ITPK1 continues this trend.

### Molecular modelling identifies substrate specificity pockets and highlights roles of tab and tether insertions in determining hydroxy-kinase specificity

**Figure 5:**
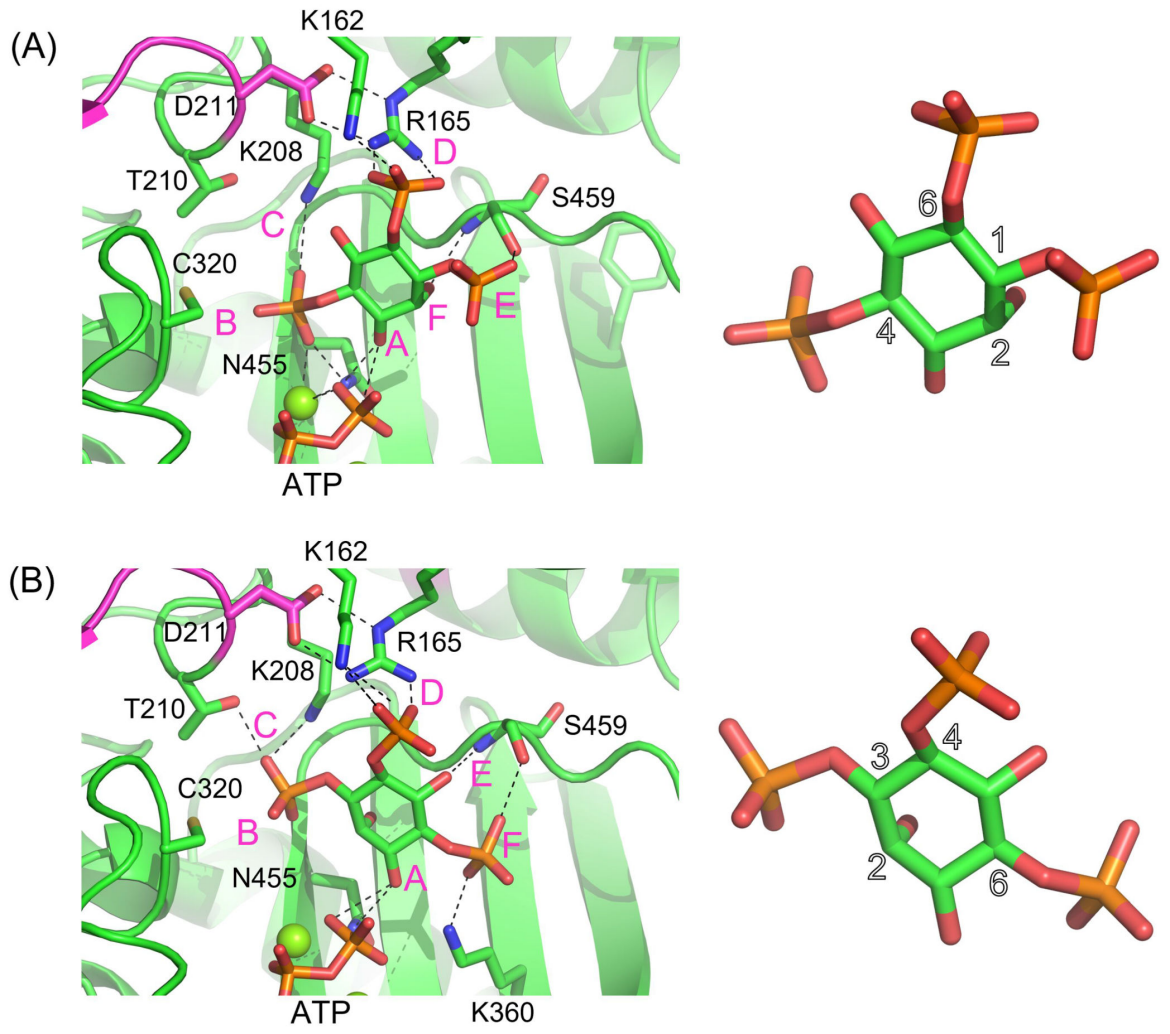
Prediction of the binding modes of the enantiomeric substrate pair Ins(1,4,6)P_3_ and Ins(3,4,6)P_3_ to the active site of *At*ITPK4. **(A) Left panel -** Closeup view of the energy minimized predicted binding mode of the good substrate, Ins(1,4,6)P_3_, in the kinase domain active site. Enzyme shown in cartoon format and coloured green except for the residues of the tab insertion which are coloured magenta. The substrate and active site residues (labelled) with which it forms polar interactions are shown in stick format with carbon coloured green, oxygen red, nitrogen blue and phosphorus orange. Polar interactions are indicated by dashed lines. Specificity subsites are labelled A-F such that the hydroxyl group positioned to accept the γ-phosphate of ATP by in-line transfer (the hydroxyl attached to carbon 3 of the inositol ring, in this case) occupies subsite A and the remaining subsites are arrayed in a clockwise sense when observed from the viewpoint adopted in this figure. **Right panel –** closeup of the docked Ins(1,4,6)P_3_ substrate with a selection of the carbon atom positions of the inositol ring numbered. **(B) Left panel -** View of the energy minimized predicted binding mode of the poor substrate, Ins(3,4,6)P_3_, in the kinase domain active site. The hydroxyl (attached to carbon 1 of the inositol ring, in this case) occupies subsite A. Display format and colouring as in panel (A). **Right panel –** closeup of the docked Ins(3,4,6)P_3_ substrate with a selection of the carbon atom positions of the inositol ring numbered.

The structures of substrates and products of *At*ITPK4 are shown (Figure S2). The crystal structure of *At*ITPK4 lacks a bound inositide substrate and so attempts were made through molecular modelling to predict the likely residue composition of specificity pockets. By so doing it was hoped to provide insights to the possible roles of the tab and tether insertions on substrate specificity. Consequently, models of the complexes formed by *At*ITPK4 with the inositol trisphosphates Ins(1,4,6)P_3_ or Ins(3,4,6)P_3_ (Figure 5) and with the tetraphosphates Ins(1,4,5,6)P_4_ or Ins(3,4,5,6)P_4_ (Figure S9) were constructed and relaxed by energy minimization. Critically, these potential substrates were manually docked to the active site of *AtI*TPK4 and oriented and positioned for stereochemically-favoured hydroxy-kinase action at the C3 or C1 hydroxyl of the substrate, as appropriate [41]. The consequence of this constraint is that, if the two faces of the inositol ring of the substrate are termed obverse and reverse (where the obverse face is viewed looking down onto the unique axial phosphate at the 2-carbon position), then for enzymatic phosphorylation at the 1-, 3- or 5-hydroxyl positions of the ring, the reverse face of the ring should be oriented towards an observer sitting on the γ-phosphate of the ATP coenzyme. Conversely, the obverse orientation will be favoured for phosphorylation at the 4- or 6-hydroxyl positions and indeed this is what is observed in the complex of *Eh*ITPK1 with Ins(1,3,4)P_3_ and the non-hydrolyzable ATP analogue, AMP-PCP [34]. While the resulting molecular models of the complexes are speculative and should be interpreted with caution, they do at least suggest features of the active site of *At*ITPK4 which may help provide a molecular context for the observed substrate preference of the enzyme.

To best describe the predicted differential interactions of the enzyme with enantiomeric substrate pairs, we adopt the specificity subsite nomenclature of Miller *et al.* [34]. In this scheme, subsite A is the site of phosphoryl transfer and constitutes the catalytic centre. From a vantage point positioned on the γ-phosphate of ATP looking at the reverse face of the substrate, specificity subsites available to bind phosphates attached at the inositol ring carbon positions are then labelled B-F in a clockwise fashion, following the order of increasing carbon number of the *myo-*inositol ring (Figure 5, Figure S9). The residues contributing to the specificity subsites in the relaxed models of the inositol tris- and tetrakisphosphates with *At*ITPK4 are summarized in (Table S1). Residues of the N-terminal domain tab insertion contribute principally to specificity pockets C and D. Conserved in ITPK4-like sequences, residue Cys320 in the central domain contributes to pocket B. This residue is replaced by an aspartate in *Zm*ITPK1 and *At*ITPK1. On the other hand, specificity pockets E and F are underpopulated in ITPK4s due to the absence of the insertion in the tether polypeptide. The tether insertion in the central domain of ITPK1s contributes residues to specificity pockets E and F, as seen, for example, in the structures of the complexes of *Eh*ITPK1 with Ins(1,3,4)P_3_ and Ins(1,3,4,6)P_4_ (PDB entries 1Z2P and 1Z2O, respectively). In plant ITPK1 an asparagine residue (Asn280 in *Zm*ITPK1 and Asn271 in *At*ITPK1) contributes to the F-pocket. This residue becomes a glycine in ITPK4s (Gly437 in AtITPK4). Potential poses that would allow phosphorylation of Ins(1,3,4)P_3_ on the 5-hydroxyl and Ins(3,4,5)P_3_ on the 6-hydroxyl (Table 1) are illustrated (Figure S10), with the latter requiring obverse orientation of the substrate.

The predicted productive binding poses (i.e. required for hydroxy-kinase activity) of the poor substrates Ins(3,4,6)P_3_ and Ins(3,4,5,6)P_4_ with *At*ITPK4 notably lack a phosphate in the Bsubsite. Indeed, phosphorylation may be disfavoured by the poorer solvation of the axial 2-hydroxyl group of the substrate found in this pocket. Conversely, the 4-phosphate in this pocket as found in the predicted complexes with the better substrates Ins(1,4,6)P_3_ (Figure 5) and Ins(1,4,5,6)P_4_ (Figure S9) is predicted to make a hydrogen bond with the thiol group of Cys320 and also coordinate a magnesium ion. If accurately predicted, these interactions are likely crucial for specific hydroxy-kinase activity by *At*ITPK4. The arguments we have used to predict the docking poses of potential inositol phosphate substrates to *At*ITPK4 will also be applicable to *At*ITPK1. It follows that the predicted docking poses of Ins(3,4,6)P_3_ and Ins(3,4,5,6)P_4_ to *At*ITPK1 will result in occupation of the F-subsite by the 6-phosphate of the substrate. In *Zm*ITPK1s this phosphate is predicted to interact with the sidechain of an Asn280. The presence of Gly437 at this site in *At*ITPK4 and the absence of the tether insertion may help to explain the poor activity of this enzyme towards these potential substrates.

### *At*ITPK4 does not phosphorylate Ins(1,2,3,4,5)P_5_

It is tempting to compare the crystal structures of InsP_6_-coordinated *Zm*ITPK1 H192A variant (PDB entry 7TN8) and ATP-coordinated *At*ITPK4 (this study; PDB entry 7PUP) to investigate the documented lack of InsP_6_-kinase activity of *At*ITPK4 [1,8]. However, the usefulness of the comparison is additionally limited by the paucity of information relating to the inositol phosphate hydroxy-kinase activity of *Zm*ITPK1 [33]. Nevertheless, we note that among substrates tested in the first report of this enzyme, these did not include InsP_6_ or enantiomeric pairs, Ins(3,4,5,6)P_4_ was the best substrate [42]. The product of *Zm*ITPK1-catalyzed phosphorylation of InsP_6_ is reported to be 5-InsP_7_ by virtue of it being a substrate for DIPP1 (Figure 1. [33]). We note, however, that the DIPP1-coupled phosphate release ‘pyrophosphatase’ assay indicated phosphorylation of all six InsP_5_s, while a specific 3-pyrophosphorylation of Ins(1,2,3,4,5)P_5_ was reported. Set against this remarkably promiscuous apparent pyrophosphate-synthesizing ability of *Zm*ITPK1, the inability of AtITPK4 to phosphorylate InsP_5_ isomers is a significant departure, potentially explained by the replacement of the 5-phosphate-coordinating asparagine residue (Asn280) of *Zm*ITPK1 with a glycine residue (Gly437) in *At*ITPK4. The remaining 5-phosphate-coordinating residues in the *Zm*ITPK1 structure, Lys198, Tyr200 and Lys306 are conserved in AtITPK4. However, without structures for Ins(1,2,3,4,5)P_5_-coordinated *Zm*ITPK1 or *At*ITPK4, the explanation of apparent different pyrophosphate products awaits further structural studies.

### AtITPK4 shares the reversibility of phosphotransferase activity of ITPK1

The absence of coordinated nucleotide in crystal structures of plant ITPKs compromises molecular interpretation of the broad range of phosphotransfer catalyzed by this family of enzymes. Nonetheless, we tested the ability of *At*ITPK4 to execute phosphotransfer to ADP using the HPLC-based assay of Whitfield et al. [9]. Dual UV detection at 290 nm and 254 nm detects inositol phosphate (as complex with post-column ferric ion) at 290 nm and simultaneously detects nucleotide at 254 nm. HPLC analysis of products of assays in which *At*ITPK4 was incubated with inositol phosphate substrate and ADP overnight showed that *At*ITPK4 most efficiently uses Ins(1,3,4,6)P_4_ (the product of its preferred kinase substrate) in a phosphotransferase reaction to ADP (Table 1). An ADP-dependent phosphatase activity against Ins(1,3,4,5,6)P_5_ was reported for *Zm*ITPK1 [33], but neither a nucleotide nor specific InsP_4_ product were identified. Nonetheless, the precedent of earlier work on *Hs*ITPK1 [43], *At*ITPK1 and other plant ITPKs [25] [26] [28] [29] make it likely that the *Zm*ITPK1 product is Ins(3,4,5,6)P_4_. *At*ITPK4 also showed ATP-generating phosphotransferase activity with Ins(1,3,4)P_3_, Ins(1,4,6)P_3_, Ins(1,4,5,6)P_4_ and Ins(1,3,4,6)P_4_ (Table 1). For the latter substrate, it is unlikely that the product is a racemic mixture of Ins(1,4,6)P_3_/Ins(3,4,6)P_3_ (the two are unresolvable) but rather a mixture of unequal amounts – because in the forward direction the Ins(1,4,6)P_3_ enantiomer is preferred.

### Nucleotide binding in the plant ITPK family

The reversal of enantiospecificity for inositol phosphate hydroxy-kinase activities of AtITPK4 (this study) compared with *At*ITPK1 [9] led us to revisit potential phosphotransfer (including phosphatase) reactions – here with InsP_3_ and InsP_4_ substrates. *At*ITPK4 shows much lower inositol phosphate hydroxy-kinase activity (several orders of magnitude) than *At*ITPK1, like *At*ITPK1 [9] it shows phosphotransfer to ADP (Table 1) and in the absence of inositol phosphate *At*ITPK4 has lower phosphatase activity than *At*ITPK1 (Figure S11). These reactions are afforded a structural perspective through our crystal structure of ATP-coordinated *At*ITPK4. Unlike in the structure of *Zm*ITPK1, the nucleotide-binding region of the ATP Grasp fold of *At*ITPK4 closely resembles that seen in *Hs*ITPK1 and *Eh*ITPK1 and is formed from two 4-stranded anti-parallel β-sheets. The equivalent pattern in *Zm*ITPK1 is four plus three, where the edge strand of the sheet in the central domain is unresolved. This missing strand is found as β20 in *At*ITPK4 (Figure 4) and corresponds spatially to β12 in *Hs*ITPK1 and *Eh*ITPK1. Residues of the polypeptide following β20 (or its equivalent) form specific interactions with bound ATP in *At*ITPK4 (Figure S12) and in the human and entamoeba ITPK1s. The presence of disordered residues in *Zm*ITPK1, particularly the subset equivalent to those coordinating ATP in *At*ITPK4, is consistent with the absence of bound nucleotide in the structure of the maize enzyme. The authors [33] speculate that the density-lacking residues of *Zm*ITPK1 constitute a hinge and catalytic specificity element that somehow optimizes InsP_6_ kinase activity. While the equivalent of *Zm*ITPK1 His192, first described as nucleotide coordinating residue in *Eh*ITPK1 and *Hs*ITPK1 [34], also contacts nucleotide in *At*ITPK4, in H192A-mutated *Zm*ITPK1 the mutated residue and its neighbours adopt poses facing away from the nucleotide as it is bound in *At*ITPK4.

### A haloacid dehydrogenase (HAD)-like N-terminal domain of the ITPK4 family

Among the ITPK family in Arabidopsis, *At*ITPK4 is the only member with an N-terminal domain of approximately 150 residues preceding the kinase domain. Searches of the PDB revealed structural similarity of this domain to a broad range of haloacid dehydrogenase superfamily (HAD) proteins. A family of phosphohydrolases found in all organisms, HAD domain-containing proteins act on a broad variety of metabolites including nucleotides, sugars and phosphorylated amino acids. The function of this domain in *At*ITPK4 is unknown. Using as query the full-length amino acid sequence of *At*ITPK4, a search against non-redundant sequences revealed this domain to be found only in ITPK4 proteins from plants. That this domain is present in a broad range of species including soybean, oilseed rape and castor bean (Figure S13) suggests that it may have a regulatory or catalytic function.

The *At*ITPK4 HAD-like domain is formed from a 3-layered α/β sandwich with repeating β-α units adopting the Rossmannoid topology characteristic of the HAD superfamily (HADSF) [44]. While the minimal canonical HAD domain has a parallel central β-sheet of five strands in the order 54123, the parallel sheet of *At*ITPK4 has a sixth peripheral β-strand in order 654123 which we refer to as S1-S6 (Figure S5). HADSF proteins characteristically harbour the so-called squiggle and flap structural signatures that allow the enzyme to adopt distinct conformational states and that contribute to substrate specificity. The squiggle comprises a nearly complete single helical turn immediately following S1, whilst the flap is formed from a β-hairpin turn downstream of the squiggle and is often, but not exclusively, formed by two strands projecting from the core of the domain. The *At*ITPK4 HAD-like domain possesses the first of these but in place of the β-hairpin flap an unstructured 14 residue loop (residues 12-25 inclusive) is found placing it in the C0 family of capless HAD domains. The active site of HADSF enzymes is partly covered by the β-hairpin flap occurring after S1. Additional inserts occurring between the two strands of the flap or in the region immediately after S3 provide extensive shielding for the catalytic cavity. These inserts, termed caps, often contribute residues required for specificity or auxiliary catalytic functions and play a central role in the reactions catalyzed by most HAD hydrolases e.g. [44]. Although the β-hairpin flap feature which is responsible for substrate selectivity is absent in *At*ITPK4, the loop in this position (residues 15-22) is flexible and possibly able to fold over the active site to form a lid [44].

Despite possessing the appropriate structural characteristics of the superfamily, some sequence elements considered essential for HAD domain function are absent in *At*ITPK4 (Figure S14). The catalytic core residues are highly conserved in HADSF proteins and found in four signature motifs (Motifs I–IV) in the amino acid sequence [45]. These motifs are spatially arranged around a single binding cleft at the C-terminal end of the strands of the central sheet and form the active site of HAD superfamily enzymes. Found at the C-terminal end of strand S1, the first aspartate residue of Motif I (DxD) is required as a catalytic nucleophile. The carboxylate group of this aspartate and the backbone carbonyl of the second coordinate an Mg^2+^ cofactor. However, despite the protein being crystallized from a solution containing 100 mM MgCl_2_ no evidence for a magnesium ion was identified in electron density maps. In *At*ITPK4 this motif is replaced with the sequence DES where the aspartate and serine residues are well-conserved across ITPK4-like sequences. The absence of the second aspartate from the consensus motif sequence may not in itself preclude enzymatic activity as DehRhb, the L-haloacid dehalogenase gene from a marine member of the Rhodobacteraceae, is a functional HAD that possesses only the first aspartate residue in Motif I [46]. Motif II is found at the end of the S2 strand and contains a conserved serine or threonine residue which helps to orient the substrate for nucleophilic attack by forming a hydrogen bond with its transferring phosphoryl group. While a serine is found in *At*ITPK4 at position -1 relative to the consensus, its sidechain points in the other direction and the residue is not conserved in close homologs. Motif III centres on a conserved lysine residue found on the loop following S3 or on the helix to which this loop is connected. The function of this lysine is to stabilize the negative charge of the reaction intermediate together with Ser/Thr of motif II, however, no lysine or other basic residue is found either in this loop or on the following helix in *At*ITPK4. Finally, Motif IV maps to the loop immediately following strand S4 and contains acidic residues typically exhibiting the signature (G/S)(D/S)x3-4(D/E) (where x is any amino acid). Together with the aspartate residues of motif I, the conserved motif IV acidic Asp or Glu residues are involved in the coordination of Mg^2+^ in HADSF family members. In *At*ITPK4 the corresponding sequence is ASSRKEE but neither of the glutamate residues are oriented appropriately to serve this function, albeit we note that the quality of the electron density in this region is relatively poor. Y95, presented from strand S4, is a possible alternative, as demonstrated in the Arabidopsis VSP1 HAD domain [47].

### Searches for a catalytic activity of the HAD-like N-terminal domain of AtITPK4

Taken together, the preceding evidence suggests that the HAD domain in *At*ITPK4 is atypical. Similarity searches using DALI [48] and the coordinates of the HAD domain as query revealed a range of structural homologues. Unsurprisingly, functionally-characterized enzymes with the highest Z-scores included dehalogenases e.g. an L-2-haloacid dehalogenase (1JUD; Z=12.6) [49]. Hits were retrieved also to HAD domain proteins with verified or inferred phosphatase or phosphomutase activities e.g., a bifunctional epoxide hydrolase (4HAI; Z=10.5) [50] where the HAD domain displays lipid phosphate phosphatase activity, a β-phosphoglucomutase (4UW9; Z=10.2) [51] and a phosphoserine phosphatase (5JLP, Z=10.1) [52]. To test for catalytic activity, we sought to express and purify the separate N-terminal and C-terminal domains of *At*ITPK4. However, these efforts were unsuccessful, producing only insoluble protein. Therefore, activity was probed in the full-length enzyme alongside the close homologue, *At*ITPK1, which lacks the HAD-like domain, as a control. Tests for dehalogenase activity used chloropropionic acid revealed no activity for either protein. To determine whether the *At*ITPK4 enzyme had enzymatic properties typical of a HAD phosphatase, the enzymes were incubated with ATP, PNPP or G6P and phosphate release measured. AtITPK1 showed much greater phosphate release from ATP in the absence of inositol phosphate substrate, but neither enzyme released phosphate from G6P or PNPP in standard assay conditions for HAD proteins (Figure S11A). In the presence of inositide substrate both enzymes stoichiometrically transferred phosphate from ATP to inositide (Figure S11B, C).

## Discussion

Phylogenetic analysis identifies ITPK4 to be an outlier to the ITPK family in plants. Its possession of a HAD-like domain sets it apart from all other inositol phosphate kinases of IPK1, IP3-3K, ITPK, IPMK (IPK2) and VIH/PPIP5K classes. Whether the *At*ITPK4 HAD-like domain is functional is yet to be determined, but a lack of catalytic activity does not rule out other modulating functions, for example metabolic sensors are known to involve catalytically inactive enzymes through transcriptional activation [53]. There are few examples in the literature involving the presence of a HAD domain in addition to another functional active site. The mammalian soluble epoxide hydrolase (sEH) detoxification enzyme C terminal region contains the catalytic region responsible for its characterized activity. Also present is an N-terminal HAD phosphatase, not conserved in the plant homolog, which acts independently of the C-terminal EH activity [54]. The authors speculate that this HAD domain synergistically regulates the physiological process, perhaps by acting as a serine phosphatase to downregulate an opposing pathway.

What is clear, however, is that *itpk4* mutants that are blocked in InsP_6_, InsP_7_ and InsP_8_ synthesis lack PSR [17]. We may assume, therefore, that the activities of neither the ATP-Grasp nor HAD-like domains of *At*ITPK4 are essential for PSR, whereas the ATP-Grasp kinase activity of *At*ITPK1 is. Without a much better understanding of the inositol phosphate profile of Arabidopsis, particularly the discrimination of enantiomers, it is difficult to be categoric about the role of individual inositol phosphate species. This applies as much to inositol pyrophosphates as it does inositol phosphates. Nevertheless, we remain intrigued that the two gene families with undisputed influence on PSR (Itpk1 and Ipk1) both accumulate Ins(1,4,5,6)P_4_ and/or Ins(3,4,5,6)P_4_, that the latter enantiomer is predominant in *ipk1* [2] and, moreover, that *At*ITPK1 favours Ins(3,4,5,6)P_4_ as substrate, massively over InsP_6_. In contrast, *At*ITPK4 has a fraction of the enzyme activity of *At*ITPK1, lacks InsP_6_ kinase activity [8] and this study, shows an opposite enantiospecificity to the Ins(1,4,5,6)P_4_ / Ins(3,4,5,6)P_4_ pair, but mutants thereof have pronounced effect on InsP_6_, InsP_7_ and InsP_8_ levels without effect on PSR [6,17]. It is tempting to speculate again [9] that Ins(3,4,5,6)P_4_ is coupled metabolically through *At*IPK1 to control PSR. Whether inositol pyrophosphates and the VIH enzymes that make InsP_8_ [3,7] are the exclusive agents of PSR is coming under increased scrutiny [15], since as reported by others [6] *vih* mutants do not necessarily show PSR.

These data point to distinct roles of *At*ITPK1 and *At*ITPK4, reflected also in substantive differences in kinetic parameters. *At*ITPK1 *K*_m_ displays ATP in the range 0.52-1.22 mM [1,9], while *At*ITPK4 displays *K*_m_ ATP of 0.052 mM (this study). Even in phosphate deprived plants, it seems likely that ITPK4 is saturated with ATP - since phosphate starvation only reduces adenine nucleotide levels by a factor of two [1]. The levels of adenine nucleotides are quite poorly described in plants, though the work of Straube and coworkers [55] estimates ATP levels at 750 µM (approx. 120 nmol. g^-1^, f. wt) in rosette leaves of 33d-old Arabidopsis, an order of magnitude greater than that reported for hydroponic grown Arabidopsis [1]. An exhaustive study of metabolite levels in eight *Flaveria* species varying in C3 to C4 photosynthetic character measured ATP between 71 and 154 nmol. g^-1^ f. wt [56]. These data suggest that *At*ITPK4 activity is metabolically isolated, protected from excursions in nucleotide levels that might be expected to influence inositol pyrophosphate levels through effect on ITPK1 and IPK1 and VIH2 [1,31,33] and VIH1/2 [4,10]. Put another way, much of cellular InsP_8_ synthesis, that arising from *At*ITPK4 activity, is likely protected from excursions in energy charge. One counter argument requires compartmentation of inositol phosphate and inositol pyrophosphate synthesis, as invoked [1] to explain the altered inositol phosphate and inositol pyrophosphate profile of mutants of the InsP_6_ transporter mrp5 [57]. But, again, *mrp5* does not show PSR [1,6]. We speculate that ITPK4 retains its function in nucleotide-compromising physiological situations.

Whatever the contribution of ITPK4 to plant physiology, it is ancestral. Both ITPK1 and ITPK4 have orthologs in ancestral aquatic vascular plants. One estimate for the stem age of Lemnaceae is ca. 103.6 Ma [58]. ITPK1 and ITPK4 also have homologs in the liverwort *Marchantia polymorpha*, an ancestral terrestrial plant [59]. One estimate places divergence of the crown group (of *Marchantia polymorpha*) comprising the Ricciaceae and Oxymitraceae at ca. 115 Ma [60]. Study of the duckweed taxa identified pathways of InsP_6_ synthesis from inositol that are lipid-independent [22,23,61], proceeding from inositol and/or (via) Ins3P and Ins(3,4,5,6)P_4_, the preferred substrate of ITPK1 [9]. Others have shown that ITPK1 enzymes accept inositol monophosphates as substrates [62]. As we have shown, ITPK4 shows opposite enantiospecificity in choice of InsP_3_ and InsP_4_ substrates compared to ITPK1. Since ITPK4 lacks activity against inositol monophosphate, it seems likely that the contribution of ITPK4 to InsP_6_ and inositol pyrophosphate synthesis is epistatic to inositol monophosphate production.

## Methods

### Reagents

Inositol phosphates and assay reagents for kinase assays were obtained from commercial sources described [9], or, for Ins(1,4,6)P_3_/Ins(3,4,6)P_3_ and Ins(1,4,5,6)P_4_/Ins(3,4,5,6)P_4_ enantiomeric pairs, as described (Figure S2, S3).

### Protein purification

AtITPK4 was cloned essentially as described for *At*ITPK1 [9] (except that a pOPINE rather than pOPINF plasmid was employed. The primers used were 5’-*AGGAGATATACCATG*AAAGGGGTTCTACTTGACGA-3’ and 5’- *GTGATGGTGATGTTT*ATGCTTCTCTTGGACAT -3’ (where italics denote the pOPINE specific sequence required for recombination). Purification was also carried out according to the previously published protocol [9] but without 3C cleavage and additional Ni-NTA affinity purification steps since the C-terminal 6×His tag is not cleavable in pOPINE constructs.

### X-ray crystal structure determination

Purified *At*ITPK4 was concentrated to 10 mg/ml and single crystals grown using the sitting drop vapour diffusion method by equilibration at 16 °C against a crystallization solution containing 20% PEG 4000, 0.1 M MgCl_2_, 0.1 M Tris.HCl pH 7.8. A single crystal was harvested into a cryoprotect solution containing 30 % (v/v) ethylene glycol and X-ray diffraction data collected at 100 °C on beamline I04 at the Diamond Light Source (Oxford). Molecular replacement phasing was performed with Phaser [63] and the crystal structure of *Entamoeba histolytica* inositol 1,3,4-trisphosphate 5/6-kinase in complex with ADP and Mg^2+^ (PDB entry 1Z2N) [34] as a search model. Extensive manual rebuilding using Coot [64] interspersed with restrained refinement with Phenix.refine [63,65,66] as necessary to complete the final structure including the 150 amino acid N-terminal domain not present in the molecular replacement search model. Refinement employed a TLS model generated with the TLSMD web server [67].

### Protein structure and sequence analysis

The predicted structure model for *At*ITPK1 was taken from the AlphaFold Protein Structure Database (AlphaFold DB, https://alphafold.ebi.ac.uk) [38,39]. Structure-based sequence alignments were calculated using POSA (Partial Order Structure Alignment) [68,69]. Other sequence alignments were performed using Cobalt [70]. Identification of ITPK4-like sequences from the UNIREF90 database, their alignment and subsequent derivation of estimates of amino acid conservation were carried out using CONSURF [71]. The list of homologs was manually edited to retain only those which spanned at least 95 % of the amino acid sequence of *At*ITPK4 and for which the expectation value (E-value) was less than or equal to 1x10^-130^. A total of 95 such sequences were retained. Position-specific conservation scores were then computed using a Bayesian algorithm [72] and these scores divided into a discrete scale of nine grades for visualization.

### Molecular modelling

Molecular models of the complex between inositol tris- and tetrakisphosphates with *At*ITPK4 were generated with reference to the crystal structures of *Eh*ITPK1 in complex with Mg^2+^/AMP-PCP/Ins(1,3,4)P_3_ (PDB entry 1Z2P) and with Mg^2+^/ADP/Ins(1,3,4,6)P_4_ (PDB entry 1Z2O), respectively. Least squares superposition of the β-sheet residues of the C-terminal domains of *Eh*ITPK1 from PDB entry 1Z2P and AtITPK4 produced, by direct transfer of atomic coordinates, a draft docking pose of Ins(1,3,4)P_3_ along with ATP and two magnesium ions to the *At*ITPK4 crystal structure. Subsequent *in silico* substitution of phosphate groups of the inositol polyphosphate ligand generated models of, in turn, Ins(1,4,6)P_3_ or Ins(3,4,6)P_3_. Rotation of the ligands 180° about the C2-C5 axis (were Cn indicates the carbon number, n, of the inositol ring) followed by rotation about an axis normal to the ring produced models of Ins(1,4,6)P_3_ or Ins(3,4,6)P_3_ bound to *At*ITPK4 positioned for stereochemically-favoured hydroxy-kinase action at the C3 or C1 hydroxyl of the substrate, respectively [37]. A similar process involving PDB entry 1Z2O produced models of Ins(1,4,5,6)P_4_ or Ins(3,4,5,6)P_4_ along with ATP and magnesium ions docked to *At*ITPK4. All models of the complexes were subsequently energy minimized to convergence using the Prime module of Schrodinger (Schrödinger Release 2022-2: Prime, Schrödinger, LLC, New York, NY, 2021) employing the all-atom OPLS force field [73,74]. Conserved water molecules identified in high resolution crystal structures have been frequently linked to enzyme function [75,76] but their positions are unreliable at the resolution of the crystal structure of *At*ITPK4 and so they were discarded and the VSGB 2.1 implicit solvent model [77] employed. Illustrations were generated using the PyMOL Molecular Graphics System, Version 2.5 (Schrödinger, LLC).

### HPLC assays

Both Standard and ATP Regeneration assays were performed in 20 mM HEPES pH 7.3, 6 mM MgCl_2_, 10 mM LiCl_2_ and 1 mM DTT. Regeneration assays contained 5 mM phosphocreatine, 3 U creatine kinase and 1 mM ATP [9]. To identify substrates of *At*ITPK4, regeneration assays were incubated overnight with 3 μM *At*ITPK4. Specifically, for kinetic comparisons of Ins(1,4,6)P_3_, Ins(3,4,6)P_3_ and Ins(1,4)P_2_, regeneration assays were set up as described using 3 μM AtITPK4 and incubated for 20 mins at 25 °C. For determination of *K*_m_ and *V*_max_, 3 μM *At*ITPK4 was incubated with 1 mM Ins(1,4,6)P_3_ and ATP at 0-5 mM for 1 hour at 25 °C. For ATP phosphatase activity assays, 1 mM ATP with or without 1 mM substrate was incubated with 10 μM *At*ITPK1 or *At*ITPK4 at 25 °C for 2 hours. HPLC analysis and detection of inositol phosphates by complexation with ferric ion was performed as described [9]. Data were analysed using GraphPad software (GraphPad Software Inc., San Diego, USA) with Michaelis-Menten or substrate inhibition fit.

### HPLC-ICP-MS

Whole soil-grown plants (triplicates) of Col0, *itpk4-1*, an ITPK4 overexpression line and a single ITPK4-YFP- complemented *itpk4-1* plant (ITPK4-OE) [5] were frozen in LN_2,_ ground in a mortar and pestle and extracted with 1 M perchloric acid. The extract was treated with TiO_2_ [78], recovered in 300 μL water and aliquots injected onto a CarboPac PA200 column. Inositol phosphates were eluted with a gradient of HCl [9] and detected as PO^+^, m/z 47, using a Thermo Icap-TQ (Thermo Scientific) triple quadrupole Inductively Coupled Plasma-Mass Spectrometer (ICP-MS) used as a HPLC detector.

### Phosphate release assays

Performed as described [9] using 1 mM substrate (ATP, PNPP or G6P) and 10μM *At*ITPK4 or *At*ITPK1.

### HAD assay with chloropropionic acid

A colorimetric assay was performed as described by Hou et al. [79] with *At*ITPK4 concentrations 0 µM, 1 µM, 2 µM and 4 µM. Additionally NaCl, MgCl_2_ and LiCl_2_ were tested for effects on activity. Assays were monitored at 540 nm for 1 hour and then a single plate read performed after overnight incubation.

## Supplementary Material

Supplementary Material

## Figures and Tables

**Table 1:** Reactions catalyzed by AtITPK4

Kinase reaction			
		Assay type	
Substrate	Product	Regeneration	Standard
InsIP		-	-
Ins3P			-
			
Ins(1,4)P_2_	Ins(1,3,4)P_3_	+ +	**
			
Ins(1,3,4)P_3_	Ins(1,3,4,5)P_4_	+	*
			
Ins(1,4,5)P_3_			-
			
Ins(1,4,6)P_3_	Ins(1,3,4,6)P_4_	++ +	***
Ins(3,4,6)P_3_	Ins(1,3,4,6)P_4_	+	*
			
Ins(3,4,5)P_3_	Ins(3,4,5,6)P_4_		**
			
Ins(4,5,6)P_3_		-	
			
Ins(1,2,4,6)P_4_			-
Ins(2,3,4,6)P_4_			-
			
Ins(1,3,4,5)P_4_			-
Ins(1,3,5,6)P_4_			-
			
Ins(1,3,4,6)P_4_			-
			
Ins(1,4,5,6)P_4_	Ins(1,3,4,5,6)P_5_	++ +	**
Ins(3,4,5,6)P_4_			-
			
Ins(1,2,3,4,5)P_5_		-	
Ins(1,2,3,5,6)P_5_		-	
			
Ins(1,2,3,4,6)P_5_		-	
			
Ins(1,3,4,5,6)P_5_		-	
			
Ins(1,2,4,5,6)P_5_		-	
Ins(2,3,4,5,6)P_5_		-	
			
Ins(1,2,3,4,5,6)Pe		-	
			
**Phosphotransfer to ADP**			
**Substrate**	**InsP product**		
Ins(1,3,4)P_3_	Not verified	+ +	
			
Ins(1,4,6)P_3_	Not verified	+ +	
			
Ins(1,3,4,6)P_4_	Ins(1,4,6)P_3_/Ins(3,4,6)P_3_	++ +	
			
Ins(1,4,5,6)P_4_	Not verified	+	

For ATP-regeneration assays, -/+/++/+++ indicates reaction strength within pair of enantiomers or classes (InsP_3_, InsP_4_) of compounds; - indicates absence of activity; otherwise, not tested. Without ATP regeneration (standard assay), reaction indicated -/*/**/****. Not verified, InsP_2_s are poorly resolved on the column used and standards were not used. All reactions which failed to give products were analyzed beside positive controls that yielded products. Structures of substrates/products shown in Fig. S1.

**Table 2:** Data collection and refinement statistics^a^.

DATA COLLECTION	
Wavelength / Å	0.9795
Resolution range	44.17 - 1.91 (1.98 - 1.91)
Space group	P 21 21 21
Unit cell	45.62 61.8 176.47 90 90 90
Total reflections	519292 (50938)
Unique reflections	39633 (3892)
Multiplicity	13.1 (13.1)
Completeness (%)	99.90 (99.67)
Mean I/sigma(I)	10.14 (0.87)
Wilson B-factor	33.32
R-merge	0.1853 (2.965)
R-meas	0.1928 (3.084)
R-pim	0.05281 (0.8422)
CC1/2	0.998 (0.376)
CC*	1.0 (0.739)
**REFINEMENT**	
Reflections used in refinement	39622 (3887)
Reflections used for R-free	1952 (185)
R-work	0.2097 (0.3150)
R-free	0.2409 (0.3190)
CC(work)	0.957 (0.683)
CC(free)	0.933 (0.613)
Number of non-hydrogen atoms	3981
macromolecules	3733
ligands	43
solvent	217
Protein residues	479
RMS(bonds)	0.009
RMS(angles)	1.15
Ramachandran favored (%)	94.90
Ramachandran allowed (%)	4.03
Ramachandran outliers (%)	1.06
Rotamer outliers (%)	2.38
Clashscore	18.89
Average B-factor	48.44
macromolecules	48.93
ligands	26.70
solvent	43.13
Number of TLS groups	4

a Statistics for the highest-resolution shell are shown in parentheses.

## Data Availability

Coordinates and diffraction data for the crystal structure of *At*ITPK4 in complex with ATP have been deposited in the PDB with accession code 7PUP [80].

## Abbreviations

AMP-PCP: 5'-adenylyl methylenediphosphonate
*At*IPK1: *Arabidopsis thaliana* inositol pentakisphosphate 2-kinase
*At*ITPK1: *Arabidopsis thaliana* inositol tris/tetrakisphosphate kinase 1
BSTFA: bis-trifluoroacetamide
DTT: reduced dithiothreitol
EDTA: ethylenediamine tetra-acetic acid
HEPES: 4-(2-hydroxyethyl)-1-piperazineethane sulfonic acid
His: histidine
HPLC: high-pressure liquid chromatography
*Eh*ITPK1: *Entamoeba histolytica* inositol tris/tetrakisphosphate kinase 1
*Hs*ITPK1: *Homo sapiens* inositol tris/tetrakisphosphate kinase 1
IP3-3K: inositol 1,4,5-trisphosphate 3-kinase
IP6K: inositol hexakisphosphate kinase
IPMK (IPK2): inositol polyphosphate multikinase
Ins(1,3,4)P_3_: 1d-*myo*-inositol 1,3,4-trisphosphate
Ins(1,4,6)P_3_: 1d-*myo*-inositol 1,4,6-trisphosphate
Ins(3,4,6)P_3_: 1d-*myo*-inositol 3,4,6-trisphosphate
Ins(4,5,6)P_3_: *myo*-inositol 4,5,6-trisphosphate
Ins(1,2,4,6)P_3_: 1d-*myo*-inositol 1,2,4,6-tetrakisphosphate
Ins(1,3,4,6)P_4_: *myo*-inositol 1,3,4,6-tetrakisphosphate
Ins(1,4,5,6)P_4_: 1d-*myo*-inositol 1,4,5,6-tetrakisphosphate
Ins(1,3,5,6)P_4_: 1d-*myo*-inositol 1,3,5,6-tetrakisphosphate
Ins(2,3,4,6)P_4_: 1d-*myo*-inositol 2,3,4,6-tetrakisphosphate
Ins(3,4,5,6)P_4_: 1d-*myo*-inositol 3,4,5,6-tetrakisphosphate
Ins(1,2,3,4,5)P_5_: 1d-*myo*-inositol 1,2,3,4,5-pentakisphosphate
Ins(1,2,3,4,6)P_5_: *myo*-inositol 1,2,3,4,6-pentakisphosphate
Ins(1,2,3,5,6)P_5_: 1d-*myo*-inositol 1,2,3,5,6-pentakisphosphate
Ins(1,2,4,5,6)P_5_: 1d-*myo*-inositol 1,2,4,5,6-pentakisphosphate
Ins(1,3,4,5,6)P_5_: *myo*-inositol 1,3,4,5,6-pentakisphosphate
Ins(2,3,5,4,6)P_5_: 1d-*myo*-inositol 2,3,4,5,6-pentakisphosphate
InsP_6_, Ins(1,2,3,4,5,6)P_6_: *myo*-inositol 1,2,3,4,5,6-hexakisphosphate
3-PP-Ins(1,2,4,5)P_4_: 1d-3-diphospho-*myo*-inositol 1,2,4,5-tetrakisphosphate
5-PP-Ins(1,2,3,4)P_4_: 1d-5-diphospho-*myo*-inositol 1,2,3,4-tetrakisphosphate
1-InsP_7_, 1-PP-InsP_5_: 1d-1-diphospho-*myo*-inositol 2,3,4,5,6-pentakisphosphate
3-InsP_7_, 3-PP-InsP_5_: 1d-3-diphospho-*myo*-inositol 1,2,4,5,6-pentakisphosphate
4-InsP_7_, 4-PP-InsP_5_: 1d-4-diphospho-*myo*-inositol 1,3,5,6-pentakisphosphate
5-InsP_7_, 5-PP-InsP_5_: 5-diphospho-*myo*-inositol 1,2,3,4,6-pentakisphosphate
6-InsP_7_, 6-PP-InsP_5_: 1d-6-diphospho-*myo*-inositol 1,2,3,4,5-pentakisphosphate
1,5-InsP_8_: 1d-1,5-bis-diphospho-*myo*-inositol 2,3,4,6-tetrakisphosphate
LC-ICP-MS: liquid chromatography inductively coupled plasma mass spectrometry
Ni-NTA: nickel-nitriloacetic acid
PCR: polymerase chain reaction
PDB: Protein DataBank
P_i_: orthophosphate
PHR1: Arabidopsis Phosphate Starvation Response 1
PPIP5K: diphosphoinositol pentakisphosphate kinase
SPX1: SYG1/Pho81/XPR1 domain-containing protein 1
Tris: tris(hydroxymethyl)aminomethane
VIH1: *Arabidopsis thaliana* diphosphoinositol pentakisphosphate kinase 1
VIH2: *Arabidopsis thaliana* diphosphoinositol pentakisphosphate kinase 2.
*Zm*ITPK1: *Zea mays* inositol tris/tetrakisphosphate kinase 1.

## References

[1] Riemer E, Qiu D, Laha D, Harmel RK, Gaugler P, Gaugler V, Frei M, Hajirezaei MR, Laha NP, Krusenbaum L, Schneider R (2021). ITPK1 is an InsP_6_/ADP phosphotransferase that controls phosphate signaling in Arabidopsis. Mol Plant.

[2] Stevenson-Paulik J, Bastidas RJ, Chiou ST, Frye RA, York JD (2005). Generation of phytate-free seeds in Arabidopsis through disruption of inositol polyphosphate kinases. Proc Natl Acad Sci U S A.

[3] Desai M, Rangarajan P, Donahue JL, Williams SP, Land ES, Mandal MK, Phillippy BQ, Perera IY, Raboy V, Gillaspy GE (2014). Two inositol hexakisphosphate kinases drive inositol pyrophosphate synthesis in plants. Plant J.

[4] Dong J, Ma G, Sui L, Wei M, Satheesh V, Zhang R, Ge S, Li J, Zhang TE, Wittwer C, Jessen HJ (2019). Inositol pyrophosphate InsP_8_ acts as an intracellular phosphate signal in Arabidopsis. Mol Plant.

[5] Kuo HF, Chang TY, Chiang SF, Wang WD, Charng YY, Chiou TJ (2014). Arabidopsis inositol pentakisphosphate 2-kinase, AtIPK1, is required for growth and modulates phosphate homeostasis at the transcriptional level. Plant J.

[6] Kuo HF, Hsu YY, Lin WC, Chen KY, Munnik T, Brearley CA, Chiou TJ (2018). Arabidopsis inositol phosphate kinases, IPK1 and ITPK1, constitute a metabolic pathway in maintaining phosphate homeostasis. Plant J.

[7] Laha D, Johnen P, Azevedo C, Dynowski M, Weiss M, Capolicchio S, Mao H, Iven T, Steenbergen M, Freyer M, Gaugler P (2015). VIH2 regulates the synthesis of inositol pyrophosphate InsP_8_ and jasmonate-dependent defenses in Arabidopsis. Plant Cell.

[8] Laha D, Parvin N, Hofer A, Giehl RFH, Fernandez-Rebollo N, von Wiren N, Saiardi A, Jessen HJ, Schaaf G (2019). Arabidopsis ITPK1 and ITPK2 Have an Evolutionarily Conserved Phytic Acid Kinase Activity. ACS Chem Biol.

[9] Whitfield H, White G, Sprigg C, Riley AM, Potter BVL, Hemmings AM, Brearley CA (2020). An ATP-responsive metabolic cassette comprised of inositol tris/tetrakisphosphate kinase 1 (ITPK1) and inositol pentakisphosphate 2-kinase (IPK1) buffers diphosphosphoinositol phosphate levels. Biochem J.

[10] Zhu J, Lau K, Puschmann R, Harmel RK, Zhang Y, Pries V, Gaugler P, Broger L, Dutta AK, Jessen HJ, Schaaf G (2019). Two bifunctional inositol pyrophosphate kinases/phosphatases control plant phosphate homeostasis. Elife.

[11] Zhou Z, Wang Z, Lv Q, Shi J, Zhong Y, Wu P, Mao C (2015). SPX proteins regulate Pi homeostasis and signaling in different subcellular level. Plant Signal Behav.

[12] Puga MI, Mateos I, Charukesi R, Wang Z, Franco-Zorrilla JM, de Lorenzo L, Irigoyen ML, Masiero S, Bustos R, Rodriguez J, Leyva A (2014). SPX1 is a phosphate-dependent inhibitor of Phosphate Starvation Response 1 in Arabidopsis. Proc Natl Acad Sci U S A.

[13] Wild R, Gerasimaite R, Jung JY, Truffault V, Pavlovic I, Schmidt A, Saiardi A, Jessen HJ, Poirier Y, Hothorn M, Mayer A (2016). Control of eukaryotic phosphate homeostasis by inositol polyphosphate sensor domains. Science.

[14] Ried MK, Wild R, Zhu J, Pipercevic J, Sturm K, Broger L, Harmel RK, Abriata LA, Hothorn LA, Fiedler D, Hiller S (2021). Inositol pyrophosphates promote the interaction of SPX domains with the coiled-coil motif of PHR transcription factors to regulate plant phosphate homeostasis. Nat Commun.

[15] Land ES, Cridland CA, Craige B, Dye A, Hildreth SB, Helm RF, Gillaspy GE, Perera IY (2021). A Role for inositol pyrophosphates in the metabolic adaptations to low phosphate in Arabidopsis. Metabolites.

[16] Kim SI, Tai TH (2011). Identification of genes necessary for wild-type levels of seed phytic acid in *Arabidopsis thaliana* using a reverse genetics approach. Mol Genet Genomics.

[17] Wang Z, Kuo HF, Chiou TJ (2021). Intracellular phosphate sensing and regulation of phosphate transport systems in plants. Plant Physiol.

[18] Riemer E, Pullagurla NJ, Yadav R, Rana P, Jessen HJ, Kamleitner M, Schaaf G, Laha D (2022). Regulation of plant biotic interactions and abiotic stress responses by inositol polyphosphates. Front Plant Sci.

[19] Gulabani H, Goswami K, Walia Y, Roy A, Noor JJ, Ingole KD, Kasera M, Laha D, Giehl RFH, Schaaf G, Bhattacharjee S (2022). Arabidopsis inositol polyphosphate kinases IPK1 and ITPK1 modulate crosstalk between SA-dependent immunity and phosphate-starvation responses. Plant Cell Rep.

[20] Murphy AM, Otto B, Brearley CA, Carr JP, Hanke DE (2008). A role for inositol hexakisphosphate in the maintenance of basal resistance to plant pathogens. Plant J.

[21] Laha NP, Giehl RFH, Riemer E, Qiu D, Pullagurla NJ, Schneider R, Dhir YW, Yadav R, Mihiret YE, Gaugler P, Gaugler V (2022). INOSITOL (1,3,4) TRIPHOSPHATE 5/6 KINASE1-dependent inositol polyphosphates regulate auxin responses in Arabidopsis. Plant Physiol.

[22] Brearley CA, Hanke DE (1996). Inositol phosphates in the duckweed *Spirodela polyrhiza* L. Biochem J.

[23] Brearley CA, Hanke DE (1996). Metabolic evidence for the order of addition of individual phosphate esters in the myo-inositol moiety of inositol hexakisphosphate in the duckweed *Spirodela polyrhiza* L. Biochem J.

[24] Brearley CA, Hanke DE (2000). Metabolic relations of inositol 3,4,5,6-tetrakisphosphate revealed by cell permeabilization. Identification of inositol 3,4,5,6-tetrakisphosphate 1-kinase and inositol 3,4,5,6-tetrakisphosphate phosphatase activities in mesophyll cells. Plant Physiol.

[25] Sweetman D, Stavridou I, Johnson S, Green P, Caddick SE, Brearley CA (2007). *Arabidopsis thaliana* inositol 1,3,4-trisphosphate 5/6-kinase 4 (AtITPK4) is an outlier to a family of ATP-grasp fold proteins from Arabidopsis. FEBS Lett.

[26] Caddick SE, Harrison CJ, Stavridou I, Mitchell JL, Hemmings AM, Brearley CA (2008). A S*olanum tuberosum* inositol phosphate kinase (StITPK1) displaying inositol phosphate-inositol phosphate and inositol phosphate-ADP phosphotransferase activities. FEBS Lett.

[27] Chamberlain PP, Qian X, Stiles AR, Cho J, Jones DH, Lesley SA, Grabau EA, Shears SB, Spraggon G (2007). Integration of inositol phosphate signaling pathways via human ITPK1. J Biol Chem.

[28] Stiles AR, Qian X, Shears SB, Grabau EA (2008). Metabolic and signaling properties of an Itpk gene family in *Glycine max*. FEBS Lett.

[29] Josefsen L, Bohn L, Sorensen MB, Rasmussen SK (2007). Characterization of a multifunctional inositol phosphate kinase from rice and barley belonging to the ATP-grasp superfamily. Gene.

[30] Banos-Sanz JI, Sanz-Aparicio J, Whitfield H, Hamilton C, Brearley CA, Gonzalez B (2012). Conformational changes in inositol 1,3,4,5,6-pentakisphosphate 2-kinase upon substrate binding: role of N-terminal lobe and enantiomeric substrate preference. J Biol Chem.

[31] Phillippy BQ, Ullah AH, Ehrlich KC (1994). Purification and some properties of inositol 1,3,4,5,6-Pentakisphosphate 2-kinase from immature soybean seeds. J Biol Chem.

[32] Blaabjerg K, Hansen-Moller J, Poulsen HD (2010). High-performance ion chromatography method for separation and quantification of inositol phosphates in diets and digesta. J Chromatogr B Analyt Technol Biomed Life Sci.

[33] Zong G, Shears SB, Wang H (2022). Structural and catalytic analyses of the InsP_6_ kinase activities of higher plant ITPKs. FASEB J.

[34] Miller GJ, Wilson MP, Majerus PW, Hurley JH (2005). Specificity determinants in inositol polyphosphate synthesis: crystal structure of inositol 1,3,4-trisphosphate 5/6-kinase. Mol Cell.

[35] Carr S, Penfold CN, Bamford V, James R, Hemmings AM (2000). The structure of TolB, an essential component of the tol-dependent translocation system, and its protein-protein interaction with the translocation domain of colicin E9. Structure.

[36] Collins ES, Whittaker SB, Tozawa K, MacDonald C, Boetzel R, Penfold CN, Reilly A, Clayden NJ, Osborne MJ, Hemmings AM, Kleanthous C (2002). Structural dynamics of the membrane translocation domain of colicin E9 and its interaction with TolB. J Mol Biol.

[37] Daughtry KD, Huang H, Malashkevich V, Patskovsky Y, Liu W, Ramagopal U, Sauder JM, Burley SK, Almo SC, Dunaway-Mariano D, Allen KN (2013). Structural basis for the divergence of substrate specificity and biological function within HAD phosphatases in lipopolysaccharide and sialic acid biosynthesis. Biochemistry.

[38] Varadi M, Anyango S, Deshpande M, Nair S, Natassia C, Yordanova G, Yuan D, Stroe O, Wood G, Laydon A, Zidek A (2022). AlphaFold protein structure database: massively expanding the structural coverage of protein-sequence space with high-accuracy models. Nucleic Acids Res.

[39] Jumper J, Evans R, Pritzel A, Green T, Figurnov M, Ronneberger O, Tunyasuvunakool K, Bates R, Zidek A, Potapenko A, Bridgland A (2021). Highly accurate protein structure prediction with AlphaFold. Nature.

[40] Fawaz MV, Topper ME, Firestine SM (2011). The ATP-grasp enzymes. Bioorg Chem.

[41] Riley AM, Deleu S, Qian X, Mitchell J, Chung SK, Adelt S, Vogel G, Potter BVL, Shears SB (2006). On the contribution of stereochemistry to human ITPK1 specificity: Ins(1,4,5,6)P_4_ is not a physiologic substrate. FEBS Lett.

[42] Shi J, Wang H, Wu Y, Hazebroek J, Meeley RB, Ertl DS (2003). The maize low-phytic acid mutant lpa2 is caused by mutation in an inositol phosphate kinase gene. Plant Physiol.

[43] Ho MW, Yang X, Carew MA, Zhang T, Hua L, Kwon YU, Chung SK, Adelt S, Vogel G, Riley AM, Potter BVL (2002). Regulation of Ins(3,4,5,6)P(4) signaling by a reversible kinase/phosphatase. Curr Biol.

[44] Burroughs AM, Allen KN, Dunaway-Mariano D, Aravind L (2006). Evolutionary genomics of the HAD superfamily: understanding the structural adaptations and catalytic diversity in a superfamily of phosphoesterases and allied enzymes. J Mol Biol.

[45] Aravind L, Galperin MY, Koonin EV (1998). The catalytic domain of the P-type ATPase has the haloacid dehalogenase fold. Trends Biochem Sci.

[46] Novak HR, Sayer C, Isupov MN, Paszkiewicz K, Gotz D, Spragg AM, Littlechild JA (2013). Marine Rhodobacteraceae L-haloacid dehalogenase contains a novel His/Glu dyad that could activate the catalytic water. FEBS J.

[47] Chen Y, Wei J, Wang M, Shi Z, Gong W, Zhang M (2012). The crystal structure of Arabidopsis VSP1 reveals the plant class C-like phosphatase structure of the DDDD superfamily of phosphohydrolases. PLoS One.

[48] Holm L (2022). Dali server: structural unification of protein families. Nucleic Acids Res.

[49] Hisano T, Hata Y, Fujii T, Liu JQ, Kurihara T, Esaki N, Soda K (1996). Crystal structure of L-2-haloacid dehalogenase from Pseudomonas sp. YL. An alpha/beta hydrolase structure that is different from the alpha/beta hydrolase fold. J Biol Chem.

[50] Pecic S, Pakhomova S, Newcomer ME, Morisseau C, Hammock BD, Zhu Z, Rinderspacher A, Deng SX (2013). Synthesis and structure-activity relationship of piperidine-derived non-urea soluble epoxide hydrolase inhibitors. Bioorg Med Chem Lett.

[51] Park K-HJ, J-H, Park C-S, Woo E-J (2014). Structural features of archaeal β -phosphoglucomutase from hyperthermophilic Pyrococcus sp . ST04. Bio Des.

[52] Yadav GP, Shree S, Maurya R, Rai N, Singh DK, Srivastava KK, Ramachandran R (2014). Characterization of *M. tuberculosis* SerB2, an essential HAD-family phosphatase, reveals novel properties. PLoS One.

[53] Soyk S, Simkova K, Zurcher E, Luginbuhl L, Brand LH, Vaughan CK, Wanke D, Zeeman SC (2014). The enzyme-like domain of Arabidopsis nuclear beta-amylases is critical for DNA sequence recognition and transcriptional activation. Plant Cell.

[54] Cronin A, Mowbray S, Durk H, Homburg S, Fleming I, Fisslthaler B, Oesch F, Arand M (2003). The N-terminal domain of mammalian soluble epoxide hydrolase is a phosphatase. Proc Natl Acad Sci U S A.

[55] Straube H, Niehaus M, Zwittian S, Witte CP, Herde M (2021). Enhanced nucleotide analysis enables the quantification of deoxynucleotides in plants and algae revealing connections between nucleoside and deoxynucleoside metabolism. Plant Cell.

[56] Borghi GL, Arrivault S, Gunther M, Barbosa Medeiros D, Dell’Aversana E, Fusco GM, Carillo P, Ludwig M, Fernie AR, Lunn JE, Stitt M (2022). Metabolic profiles in C3, C3-C4 intermediate, C4-like, and C4 species in the genus Flaveria. J Exp Bot.

[57] Nagy R, Grob H, Weder B, Green P, Klein M, Frelet-Barrand A, Schjoerring JK, Brearley C, Martinoia E (2009). The Arabidopsis ATP-binding cassette protein AtMRP5/AtABCC5 is a high affinity inositol hexakisphosphate transporter involved in guard cell signaling and phytate storage. J Biol Chem.

[58] Tippery NP, Les DH, Cao X, Fourounjian P, Wang W (2020). The Duckweed Genomes. Compendium of Plant Genomes.

[59] Laha D, Portela-Torres P, Desfougères Y, Saiardi A (2021). Inositol phosphate kinases in the eukaryote landscape. Adv Biol Regul.

[60] Villarreal AJC, Crandall-Stotler BJ, Hart ML, Long DG, Forrest LL (2016). Divergence times and the evolution of morphological complexity in an early land plant lineage (Marchantiopsida) with a slow molecular rate. New Phytol.

[61] Bollmann O, Strother S, Hoffmann-Ostenhof O (1980). The enzymes involved in the synthesis of phytic acid in *Lemna gibba* (Studies on the biosynthesis of cyclitols, XL.). Mol Cell Biochem.

[62] Desfougères Y, Wilson MSC, Laha D, Miller GJ, Saiardi A (2019). ITPK1 mediates the lipid-independent synthesis of inositol phosphates controlled by metabolism. Proc Natl Acad Sci U S A.

[63] McCoy AJ, Grosse-Kunstleve RW, Adams PD, Winn MD, Storoni LC, Read RJ (2007). Phaser crystallographic software. J Appl Crystallogr.

[64] Emsley P, Lohkamp B, Scott WG, Cowtan K (2010). Features and development of Coot. Acta Crystallogr D Biol Crystallogr.

[65] Adams PD, Afonine PV, Bunkoczi G, Chen VB, Davis IW, Echols N, Headd JJ, Hung LW, Kapral GJ, Grosse-Kunstleve RW, McCoy AJ (2010). PHENIX: a comprehensive Python-based system for macromolecular structure solution. Acta Crystallogr D Biol Crystallogr.

[66] Afonine PV, Grosse-Kunstleve RW, Echols N, Headd JJ, Moriarty NW, Mustyakimov M, Terwilliger TC, Urzhumtsev A, Zwart PH, Adams PD (2012). Towards automated crystallographic structure refinement with phenix.refine. Acta Crystallogr D Biol Crystallogr.

[67] Painter J, Merritt EA (2006). Optimal description of a protein structure in terms of multiple groups undergoing TLS motion. Acta Crystallogr D Biol Crystallogr.

[68] Li Z, Natarajan P, Ye Y, Hrabe T, Godzik A (2014). POSA: a user-driven, interactive multiple protein structure alignment server. Nucleic Acids Res.

[69] Ye Y, Godzik A (2005). Multiple flexible structure alignment using partial order graphs. Bioinformatics.

[70] Papadopoulos JS, Agarwala R (2007). COBALT: constraint-based alignment tool for multiple protein sequences. Bioinformatics.

[71] Ashkenazy H, Abadi S, Martz E, Chay O, Mayrose I, Pupko T, Ben-Tal N (2016). ConSurf 2016: an improved methodology to estimate and visualize evolutionary conservation in macromolecules. Nucleic Acids Res.

[72] Mayrose I, Graur D, Ben-Tal N, Pupko T (2004). Comparison of site-specific rate-inference methods for protein sequences: empirical Bayesian methods are superior. Mol Biol Evol.

[73] Jorgensen WL, Tirado-Rives J (1988). The OPLS [optimized potentials for liquid simulations] potential functions for proteins, energy minimizations for crystals of cyclic peptides and crambin. J Am Chem Soc.

[74] Harder E, Damm W, Maple J, Wu C, Reboul M, Xiang JY, Wang L, Lupyan D, Dahlgren MK, Knight JL, Kaus JW (2016). OPLS3: A force field providing broad coverage of drug-like small molecules and proteins. J Chem Theory Comput.

[75] Lisgarten JN, Gupta V, Maes D, Wyns L, Zegers I, Palmer RA, Dealwis CG, Aguilar CF, Hemmings AM (1993). Structure of the crystalline complex of cytidylic acid (2'-CMP) with ribonuclease at 1.6 A resolution. Conservation of solvent sites in RNase-A high-resolution structures. Acta Crystallogr D Biol Crystallogr.

[76] Shaltiel S, Cox S, Taylor SS (1998). Conserved water molecules contribute to the extensive network of interactions at the active site of protein kinase A. Proc Natl Acad Sci U S A.

[77] Li J, Abel R, Zhu K, Cao Y, Zhao S, Friesner RA (2011). The VSGB 2.0 model: a next generation energy model for high resolution protein structure modeling. Proteins.

[78] Wilson MS, Saiardi A (2018). Inositol phosphates purification using titanium dioxide beads. Bio Protoc.

[79] Hou Z, Zhang H, Li M, Chang W (2013). Structure of 2-haloacid dehalogenase from *Pseudomonas syringae* pv. tomato DC3000. Acta Crystallogr D Biol Crystallogr.

[80] Whitfield H, He S, Brearley CA, Hemmings AM (2022). Inositol 1,3,4-trisphosphate 5/6-kinase from *Arabidopsis thaliana* (AtITPK4) in complex with ATP.

