# Supplementary material for: Diversification in the inositol tris/tetrakisphosphate kinase (ITPK) family: crystal structure and enzymology of the outlier AtITPK4
Source: Biochem J. 2023 Mar 29;480(6):433–53. doi: 10.1042/BCJ20220579 (PMC7614388; doi:10.1042/BCJ20220579)
Supplement: Supplementary Material [file BCJ-480-433-s1.pdf]

Supplementary Material

Diversification in the inositol tris/tetrakisphosphate kinase (ITPK) family: crystal structure and enzymology of the outlier *At*ITPK4

Hayley L. Whitfield[1], Sining He[1,2], Yinghong Gu[1], Colleen Sprigg[1], Hui-Fen Kuo[3], Tzyy-Jen Chiou[3], Andrew M. Riley[4], Barry V.L. Potter[4], Andrew M. Hemmings[1,5] and Charles A. Brearley[1]

[1]School of Biological Sciences, University of East Anglia, Norwich Research Park, Norwich NR4 7TJ, U.K.
[2]Department of Biology, School of Life Sciences, Southern University of Science and Technology, Nanshan, Shenzhen 518055, China.
[3]Agricultural Biotechnology Research Center, Academia Sinica, Taipei 115, Taiwan,
[4]Medicinal Chemistry & Drug Discovery, Department of Pharmacology, University of Oxford, Mansfield Road, Oxford OX1 3QT, UK
[5]College of Food Science and Technology, Shanghai Ocean University, Shanghai 201306, China.

Author for correspondence: c.brearley@uea.ac.uk
a.hemmings@uea.ac.uk

Figure S1. Inositol phosphate and ITPK involvement in the Phosphate Starvation Response.

Figure S2. Structures of substrates and products of ITPK4

Figure S3. Substrate specificity of *At*ITPK4

Figure S4. *At*ITPK4 is not an inositol hexakisphosphate kinase

Figure S5. The HAD-like domain of *At*ITPK4

Figure S6. Topology diagram of the ATP Grasp kinase domain of *At*ITPK4

Figure S7. Sequence alignment and predicted specificity subsite residues in the kinase domains of selected ITPK1s and ITPK4s from the Brassicaceae

Figure S8. Comparison of ITPK active sites

Figure S9. Prediction of the binding modes of the enantiomeric substrate pair Ins(1,4,5,6)P_4 and Ins(3,4,5,6)P_4 to the active site of *At*ITPK4

Figure S10. Prediction of the binding modes of the substrates Ins(1,3,4)P_3 and Ins(3,4,5)P_3 to the active site of AtITPK4

Figure S11. Phosphatase activities of *At*ITPK1 and *At*ITPK4

Figure S12. Interactions formed between *At*ITPK4 and bound ATP

Figure S13. Multiple Sequence Alignment of ITPK4 from plants

Figure S14. Conservation of sequence elements in homologs of the HAD domain of *At*ITPK4

Table S1. Residues contributing to the specificity subsites in the relaxed models of the inositol tris- and tetrakisphosphates with *At*ITPK4.

Figure S1. Inositol phosphate and ITPK involvement in the Phosphate Starvation Response. (A) A pathway of $InsP_6$, 5PP-$InsP_5$ (5-$InsP_7$) and 1,5-$[PP]_2$-$InsP_4$ (1,5-$InsP_8$) synthesis. (B) A model of the Phosphate Starvation Response (PSR) whereby in phosphate replete conditions interaction of inositol phosphates (pyrophosphates) with SPX1 sequester PHR1 from its activating interaction with the P1BS element of PSR genes. (C) Structures of other $InsP_7$ isomers present in plants showing symmetry relationships of enantiomeric pairs reflected in mirror plane. The enantiomerism of these in plants is not known.

Figure S2. Structures of substrates and products of ITPK4. Symmetry relationships of enantiomeric substrates (Mills and Potter, 1996; Mills et al., 2003)are presented as reflections in a mirror plane.

Figure S3. Substrate specificity of *At*ITPK4. Products of overnight reaction of *At*ITPK4 were resolved by HPLC on a CarboPac PA200 column eluted with methanesulfonic acid (black trace) beside no-enzyme controls (red trace). Substrates: (A) Ins1P, (B) Ins(1,4)P_2, (C) Ins(1,3,4)P_3, (D) Ins(1,4,6)P_3, (E) Ins(3,4,6)P_3, (F) Ins(1,4,5,6)P_4 and ATP (A-F) are indicated with respective inositol phosphate products. The source of enantiomeric substrate pairs are Mills and Potter, 1996; Mills et al., 2003. Assays such as these have been performed on at least 5 separate occasions.

Figure S4. *At*ITPK4 is not an inositol hexakisphosphate kinase. Products of 3 h (red trace) or overnight (black trace) reaction of 3 μM *At*ITPK4 with 0.5 mM substrate were resolved by HPLC on a CarboPac PA200 column eluted with HCl. (A) Ins(1,4,6)P_3, (B) $InsP_6$. Analysis of the kinase activity of *At*ITPK4 to these substrates has been performed on at least 5 separate occasions by these methods.

Figure S5. The HAD-like domain of *At*ITPK4. (A) View of a portion of the central β-sheet of the HAD-like domain of *At*ITPK4. Polypeptide is shown in cartoon representation with β-strands labelled as shown in the topology diagram (Figure 4). Residues are shown in stick format with atom colouring: carbon-green, oxygen-red, nitrogen-blue. Selected residues are labelled. The position of the squiggle (residues 9-11) is indicated. The 2Fo-Fc electron density map (grey mesh) is contoured at 1.0 σ. (B) Topology diagram of the HAD domain of *At*ITPK4. α-helices (red cylinders) and β-strands (pink arrows) are labelled and show the starting and ending residue numbers for that secondary structural element. The N-terminus is indicated by the symbol, 'N.' The single helical turn of the squiggle element is labelled.

Figure S6. Topology diagram of the ATP Grasp kinase domain of *At*ITPK4. Subdomains boxed in dark blue and labelled IIA: N-terminal domain; IIB: central domain and IIC: C-terminal domain. α-helix and β-strand secondary structural elements are labelled and show their respective starting and ending residue numbers. The tab insertion in the kinase N-terminal subdomain of *At*ITPK4 relative to the ITPK1s is enclosed in a green box. The tether polypeptide links strand β20 of the central domain to α15 of the C-terminal domain.

Figure S7. Sequence alignment and predicted specificity subsite residues in the kinase domains of selected ITPK1s and ITPK4s from the Brassicaceae. Multiple sequence alignment of enzymes from: At- *Arabidopsis thaliana* (thale cress); Aa- *Arabidopsis arenosa* (sand rock cress); Br- *Brassica rapa* (field mustard); Bo- *Brassica oleracea* (cabbage); Rs- *Raphanus sativus* (radish). Residues must be identical across all sequences to be shown with black backgrounds, while those conserved by residue class are shown with light grey backgrounds. Residues contributing to active site specificity pockets are labelled with their identifying letter (red, bold). The residues in the region of the tab and tether insertions are enclosed by magenta and blue boxes, respectively. Note that the alignments of the *At*ITPK4 and *At*ITPK1 sequences differ slightly from that shown in Figure 4. This disparity arises due to the nature of the algorithms used in each case.

Figure S8. Comparison of ITPK active sites. (**A**) *Eh*ITPK1 (1Z2P) active site surface with AMP-PCP (sticks), Mg^{2+} (spheres) and Ins(1,3,4)P_3 (sticks). (**B**) *At*ITPK4 (7PUP) active site surface with ATP (sticks) and Ins(1,3,4)P_3 (sticks) superimposed from *Eh*ITPK1 (1Z2P). (**C**) *Hs*ITPK1 active site surface with Ins(1,3,4)P_3 (sticks) superimposed from *Eh*ITPK1 (1Z2P).

Figure S9. Prediction of the binding modes of the enantiomeric substrate pair Ins(1,4,5,6)P_4 and Ins(3,4,5,6)P_4 to the active site of *At*ITPK4. (A) Left panel - Closeup view of the energy minimized predicted binding mode of the good substrate, Ins(1,4,5,6)P_4, in the kinase domain active site. Enzyme shown in cartoon format and coloured green except for the residues of the tab insertion which are coloured magenta. The substrate and active site residues (labelled) with which it forms polar interactions are shown in stick format with carbon coloured green, oxygen red, nitrogen blue and phosphorus orange. Polar interactions are indicated by dashed lines. Specificity subsites are labelled A-F such that the hydroxyl group positioned to accept the γ-phosphate of ATP by in-line transfer (the hydroxyl attached to carbon 3 of the inositol ring, in this case) occupies subsite A and the remaining subsites are arrayed in a clockwise sense when observed from the viewpoint adopted in this figure**. Right panel –** closeup of the docked Ins(1,4,5,6)P_4 substrate with the carbon atom positions of the inositol ring numbered. **(B) Left panel -** View of the energy minimized predicted binding mode of the poor substrate, Ins(3,4,5,6)P_3, in the kinase domain active site. The hydroxyl attached to carbon 1 of the inositol ring, in this case) occupies subsite A. Display format and colouring as in panel (A). **Right panel –** closeup of the docked Ins(3,4,5,6)P_4 substrate with the carbon atom positions of the inositol ring numbered.

Figure S10. Prediction of the binding modes of the substrates Ins(1,3,4)P_3 and Ins(3,4,5)P_3 to the active site of AtITPK4. (A) Left panel - Closeup view of the

predicted binding mode of the substrate, Ins(1,3,4)P_3, in the kinase domain active site. Enzyme shown in cartoon format and coloured green except for the residues of the tab insertion which are coloured magenta. The substrate and active site residues (labelled) with which it forms polar interactions are shown in stick format with carbon coloured yellow, oxygen red, nitrogen blue and phosphorus orange. Polar interactions are indicated by dashed lines. Specificity subsites are labelled A-F such that the hydroxyl group positioned to accept the γ-phosphate of ATP by in-line transfer (the hydroxyl attached to carbon 5 of the inositol ring, in this case) occupies subsite A and the remaining subsites are arrayed in a clockwise sense when observed from the viewpoint adopted in this figure. **Right panel –** closeup of the docked Ins(1,3,4)P_3 substrate with the carbon atom positions of the inositol ring numbered. **(B) Left panel -** View of the predicted binding mode of the substrate, Ins(3,4,5)P_3, in the kinase domain active site. The molecular surface of the enzyme is shown in grey revealing the active site cavity. In this case, the substrate assumes an obverse binding mode allowing the hydroxyl group attached to carbon 6 of the inositol ring to occupy subsite A. Display format and colouring as in panel (A). **Right panel –** closeup of the docked Ins(3,4,5)P_3 substrate with a carbon atom positions of the inositol ring numbered.

Figure S11. Phosphatase activities of *At*ITPK1 and *At*ITPK4. (A) Enzymes (10 μM) incubated with 1 mM glucose 6-phosphate (G6P), *para*-nitrophenol phosphate (PNPP) or ATP in the absence of inositol phosphate substrate; mean and s.d .of 4 replicates shown. Pi release was measured with molybdenum blue reagent. (B) HPLC profile of products of reaction of 10 μM *At*ITPK1 or *At*ITPK4 with 2mM ATP, measured at 254 nm. The peaks at ca. 3.5 and 9.0 min are ADP and ATP, respectively. (C) Stoichiometry of production of inositol phosphate and ADP product following reaction of *At*ITPK1 or *At*ITPK4 with their preferred substrates, Ins(3,4,5,6)P_4 (Whitfield *et al*., 2020) and Ins(1,4,6)P_3, respectively, in the presence of ATP. In the absence of inositol phosphate substrate, the production of ADP (as shown (B)) is the product of phosphatase activity. Analysis of the phosphatase activity of *At*ITPK4 to these substrates has been performed on at least 3 separate occasions by these methods.

Figure S12. Interactions formed between *At*ITPK4 and bound ATP. Hydrogen bonds shown as dashed lines. Distances in Ångstrom. Residues forming hydrophobic contacts are shown coloured gold. Figure generated by Ligplot+ (Laskowski and Swindells, 2011).

Figure S13. Multiple Sequence Alignment of ITPK4 from plants.

Figure S14. Conservation of sequence elements in homologs of the HAD domain of *At*ITPK4. The view shows the region of the HAD-like domain only and is coloured by position-specific conservation score as calculated by CONSURF (Ashkenazy *et al*., 2016). Scores are divided into a discrete scale of nine grades and projected onto the sequence alignment for visualization. The inset shows the colour scheme. The most variable positions (grade 1) are coloured turquoise, intermediately conserved positions (grade 5) are coloured white, and the most conserved positions (grade 9) coloured maroon. Green triangle- the aspartic acid of Motif I; blue triangle- Motif II; grey rectangle- Motif III; purple rectangle- Motif IV; squiggle- red rectangle; flap- yellow rectangle.

Figure S14 (continued). Conservation of sequence elements in homologs of the HAD domain of *At*ITPK4.

Table S1. Residues contributing to the specificity subsites in the relaxed models of the inositol tris- and tetrakisphosphates with *At*ITPK4.

References

Ashkenazy, H., Abadi, S., Martz, E., Chay, O., Mayrose, I., Pupko, T., Ben-Tal, N. (2016) ConSurf 2016: an improved methodology to estimate and visualize evolutionary conservation in macromolecules. Nucleic Acids Res. 44, W, W344-350.

Desai, M., Rangarajan, P., Donahue, J. L., Williams, S. P., Land, E. S., Mandal, M. K., Phillippy, B. Q., Perera, I. Y., Raboy, V., Gillaspy, G. E. (2014) Two inositol hexakisphosphate kinases drive inositol pyrophosphate synthesis in plants. Plant J. **80**(4), 642-653

Dong, J., Ma, G., Sui, L., Wei, M., Satheesh, V., Zhang, R., Ge, S., Li, J., Zhang, T. E., Wittwer, C., Jessen, H. J., Zhang, H., An, G. Y., Chao, D. Y., Liu, D. and Lei, M. (2019) Inositol Pyrophosphate $InsP_8$ Acts as an Intracellular Phosphate Signal in Arabidopsis. Mol Plant. **12**, 1463-1473

Kuo, H. F., Chang, T. Y., Chiang, S. F., Wang, W. D., Charng, Y. Y. and Chiou, T. J. (2014) Arabidopsis inositol pentakisphosphate 2-kinase, AtIPK1, is required for growth and modulates phosphate homeostasis at the transcriptional level. Plant J. **80**, 503-515

Kuo, H. F., Hsu, Y. Y., Lin, W. C., Chen, K. Y., Munnik, T., Brearley, C. A. and Chiou, T. J. (2018) Arabidopsis inositol phosphate kinases, IPK1 and ITPK1, constitute a metabolic pathway in maintaining phosphate homeostasis. Plant J. **95**(4), 613-630.

Land, E. S., Cridland, C. A., Craige, B., Dye, A., Hildreth, S. B., Helm, R. F., Gillaspy, G. E. and Perera, I. Y. (2021) A Role for Inositol Pyrophosphates in the Metabolic Adaptations to Low Phosphate in Arabidopsis. Metabolites. **11**

Laskowski, R. A., Swindells, M. B. (2011). LigPlot+: multiple ligand-protein interaction diagrams for drug discovery. J. Chem. Inf. Model., 51, 2778-2786.

Mills, S.J. and Potter, B.V.L. (1996) Synthesis of D- and L-*myo*-inositol 1,4,6-trisphosphate, regioisomers of a ubiquitous second messenger. *J Org Chem.* **61**(25):8980-8987

Mills, S.J., Riley, A.M., Liu, C., Mahon, M.F. and Potter B.V.L. (2003) A definitive synthesis of D-*myo*-inositol 1,4,5,6-tetrakisphosphate and its enantiomer D-*myo*-inositol 3,4,5,6-tetrakisphosphate from a novel butane-2,3-diacetal-protected inositol. *Chemistry* **9**(24):6207-14.

Riemer, E., Qiu, D., Laha, D., Harmel, R. K., Gaugler, P., Gaugler, V., Frei, M., Hajirezaei, M. R., Laha, N. P., Krusenbaum, L., Schneider, R., Saiardi, A., Fiedler, D., Jessen, H. J., Schaaf, G. and Giehl, R. F. H. (2021) ITPK1 is an $InsP_6$/ADP phosphotransferase that controls phosphate signaling in Arabidopsis. Mol Plant. **14**, 1864-1880

Stevenson-Paulik, J., Bastidas, R. J., Chiou, S. T., Frye, R. A. and York, J. D. (2005) Generation of phytate-free seeds in Arabidopsis through disruption of inositol polyphosphate kinases. Proc Natl Acad Sci U S A. **102**, 12612-12617

Whitfield, H., White, G., Sprigg, C., Riley, A.M., Potter, B.V.L., Hemmings, A.M., Brearley, C. A. (2020) An ATP-responsive metabolic cassette comprised of inositol tris/tetrakisphosphate kinase 1 (ITPK1) and inositol pentakisphosphate 2-kinase (IPK1) buffers diphosphoinositol phosphate levels. Biochem. J. 477, 2621-2638.

Wild, R., Gerasimaite, R., Jung, J. Y., Truffault, V., Pavlovic, I., Schmidt, A., Saiardi, A., Jessen, H. J., Poirier, Y., Hothorn, M. and Mayer, A. (2016) Control of eukaryotic phosphate homeostasis by inositol polyphosphate sensor domains. Science. **352**, 986-990

Figure S1

A A pathway of $InsP_6$, $5PP\text{-}InsP_5$ and $1,5\text{-}[PP]_2\text{-}InsP4$ synthesis

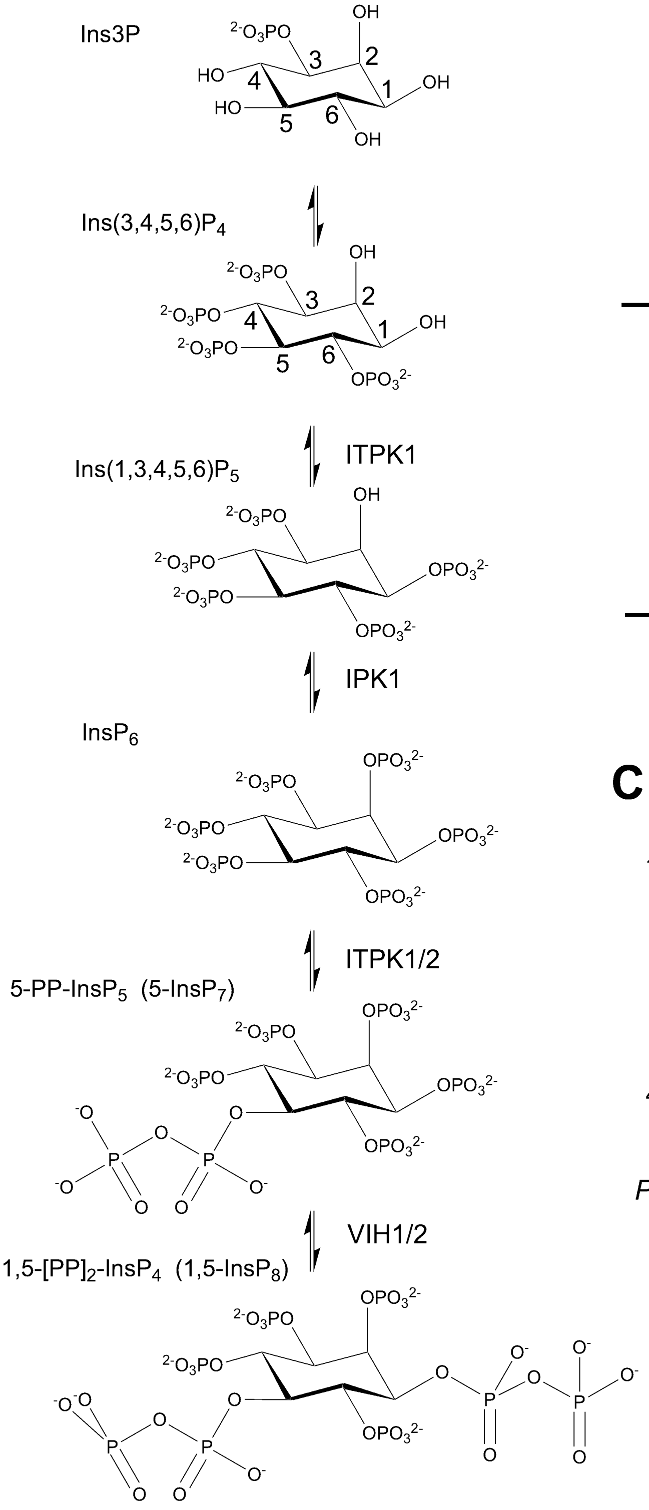

B A model of inositol phosphate involvement in phosphate starvation response after Stevenson and Paulik, 2005; Desai et al., 2014; Kuo et al., 2014; 2018; Wild et al., 2016; Dong et al., 2019; Whitfield et al., 2020; Land et al., 2021; Reimer et al., 2021; and others. *Itpk4* plants lack $InsP_6$, $PP\text{-}insP_5$ and $[PP]_2\text{-}InsP_4$, yet lack PSR; *itpk1*, *ipk1* and *vih1/2* plants show PSR to varying extent

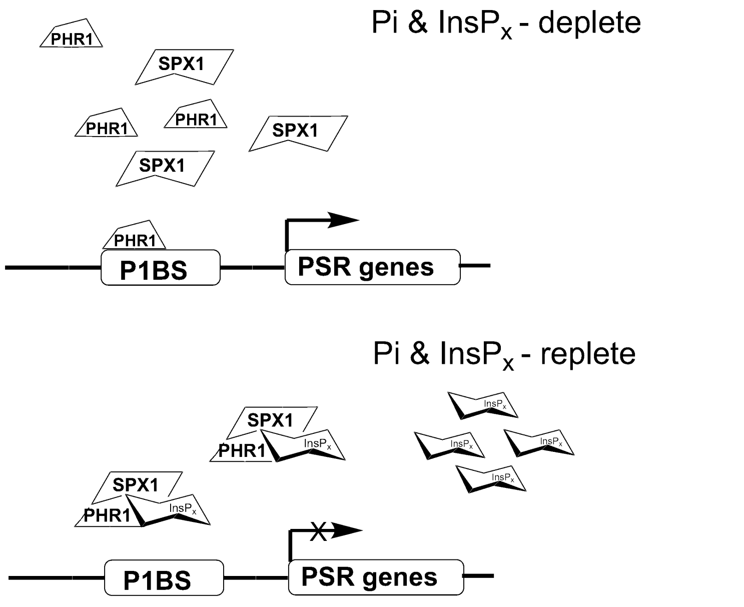

C Other PP-InsPs, enantiomers undefined, are present in plants (eg., Whitfield et al., 2020; Reimer et al., 2021)

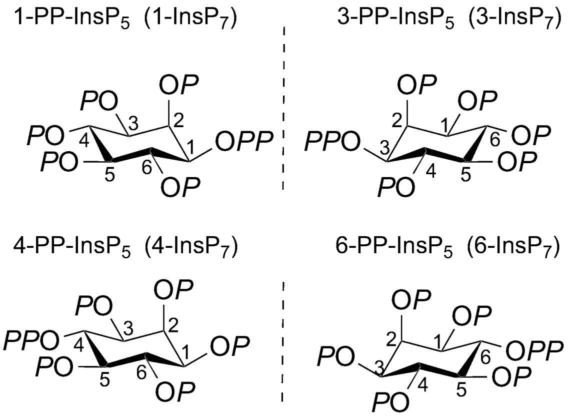

Figure S2

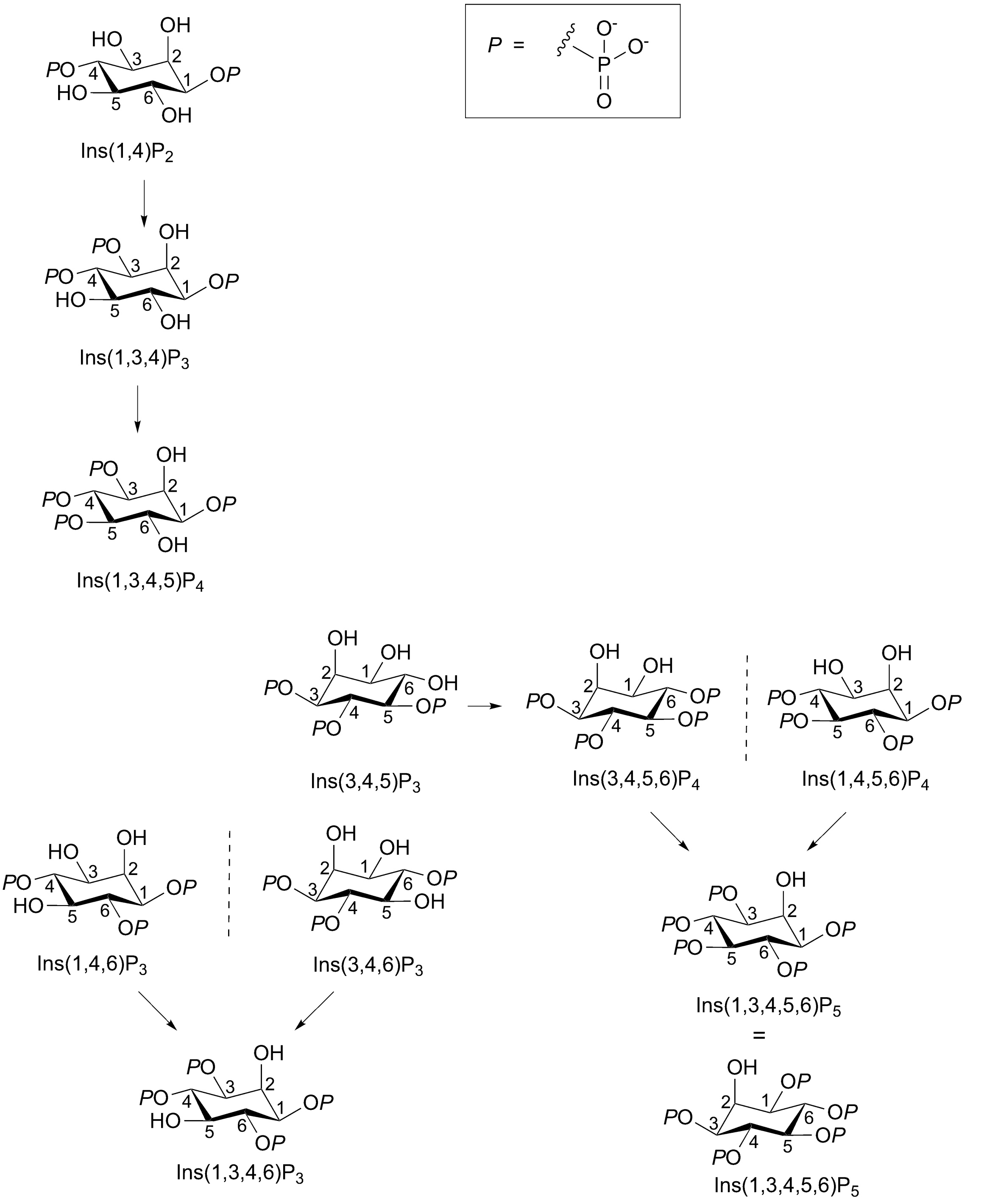
P =
Ins(1,4)P2
Ins(1,3,4)P3
Ins(1,3,4,5)P4
Ins(3,4,5)P3
Ins(3,4,5,6)P4
Ins(1,4,5,6)P4
Ins(1,4,6)P3
Ins(3,4,6)P3
Ins(1,3,4,5,6)P5
=
Ins(1,3,4,6)P3
Ins(1,3,4,5,6)P5

Figure S3

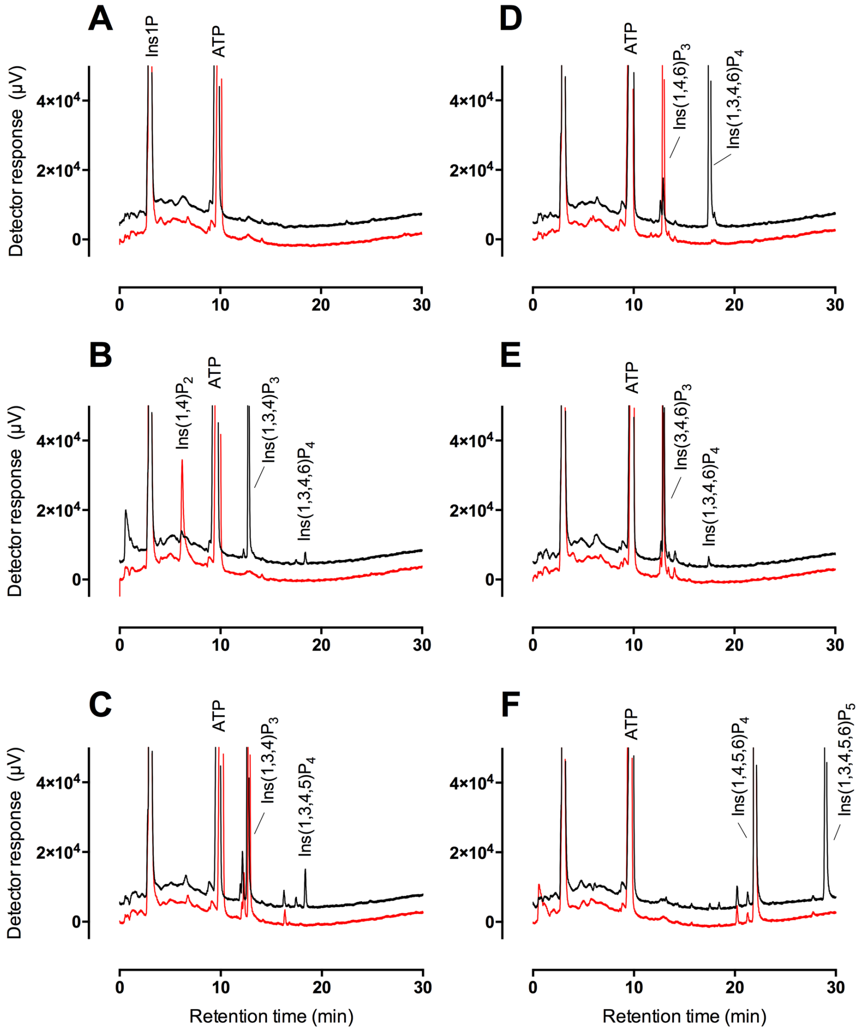
A
B
C
D
E
F
Ins1P
ATP
Ins(1,4)P2
Ins(1,3,4)P3
Ins(1,3,4,6)P4
Ins(1,3,4,5)P4
Ins(1,4,6)P3
Ins(3,4,6)P3
Ins(1,4,5,6)P4
Ins(1,3,4,5,6)P5
Detector response (µV)
4×10^4
2×10^4
0
10
20
30
Retention time (min)

Figure S4

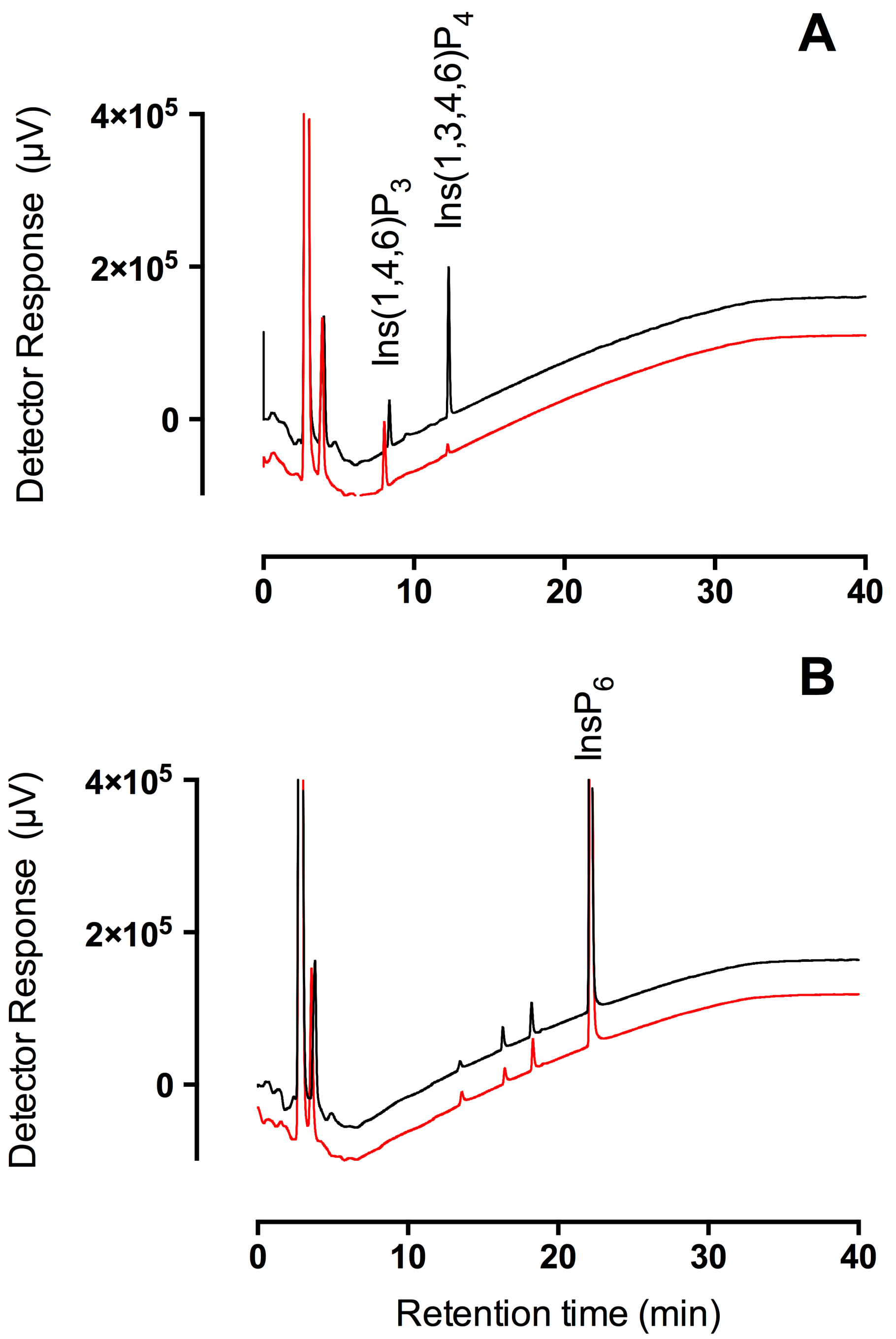
A
B
Ins(1,4,6)P3
Ins(1,3,4,6)P4
InsP6
Detector Response (µV)
4×10^5
2×10^5
0
0
10
20
30
40
Retention time (min)

Figure S5

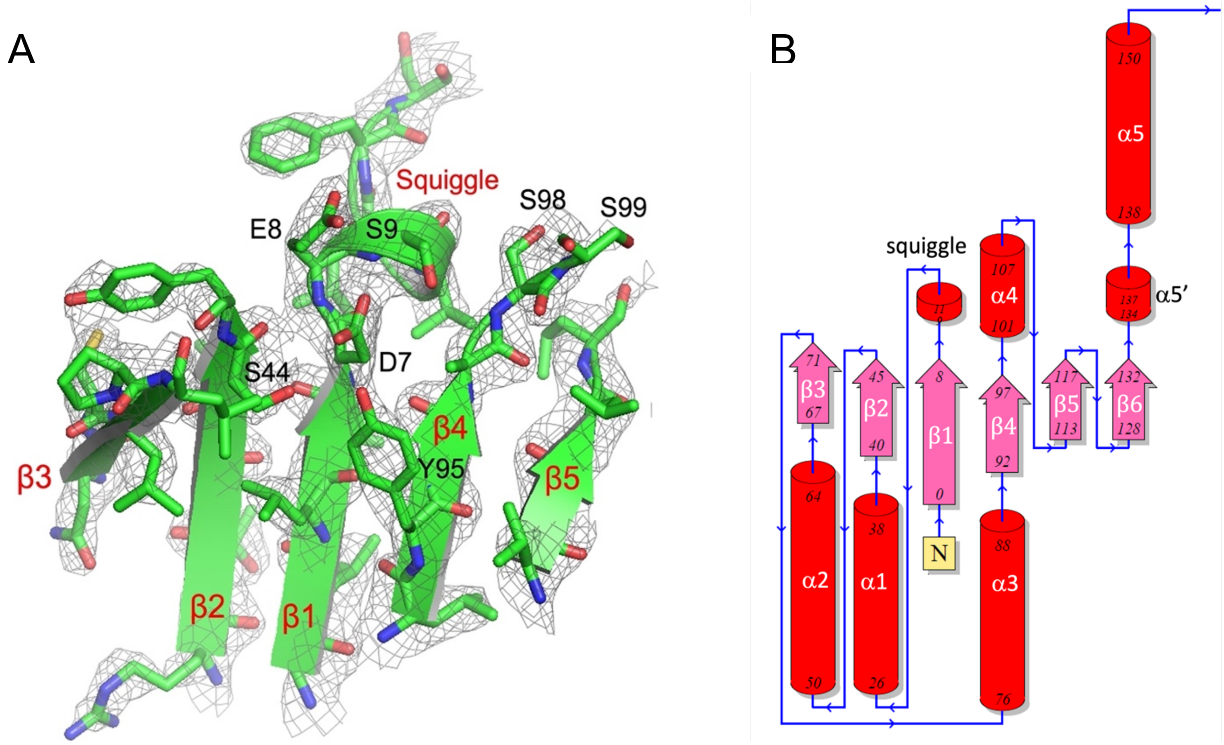
A
Squiggle
E8
S9
S98
S99
S44
D7
β4
Y95
β3
β5
β2
β1
B
squiggle
α5
150
138
α4
107
101
α5’
137
134
11
8
71
β3
67
45
β2
40
β1
0
97
β4
92
117
β5
113
132
β6
128
64
α2
50
38
α1
26
N
88
α3
76

Figure S6

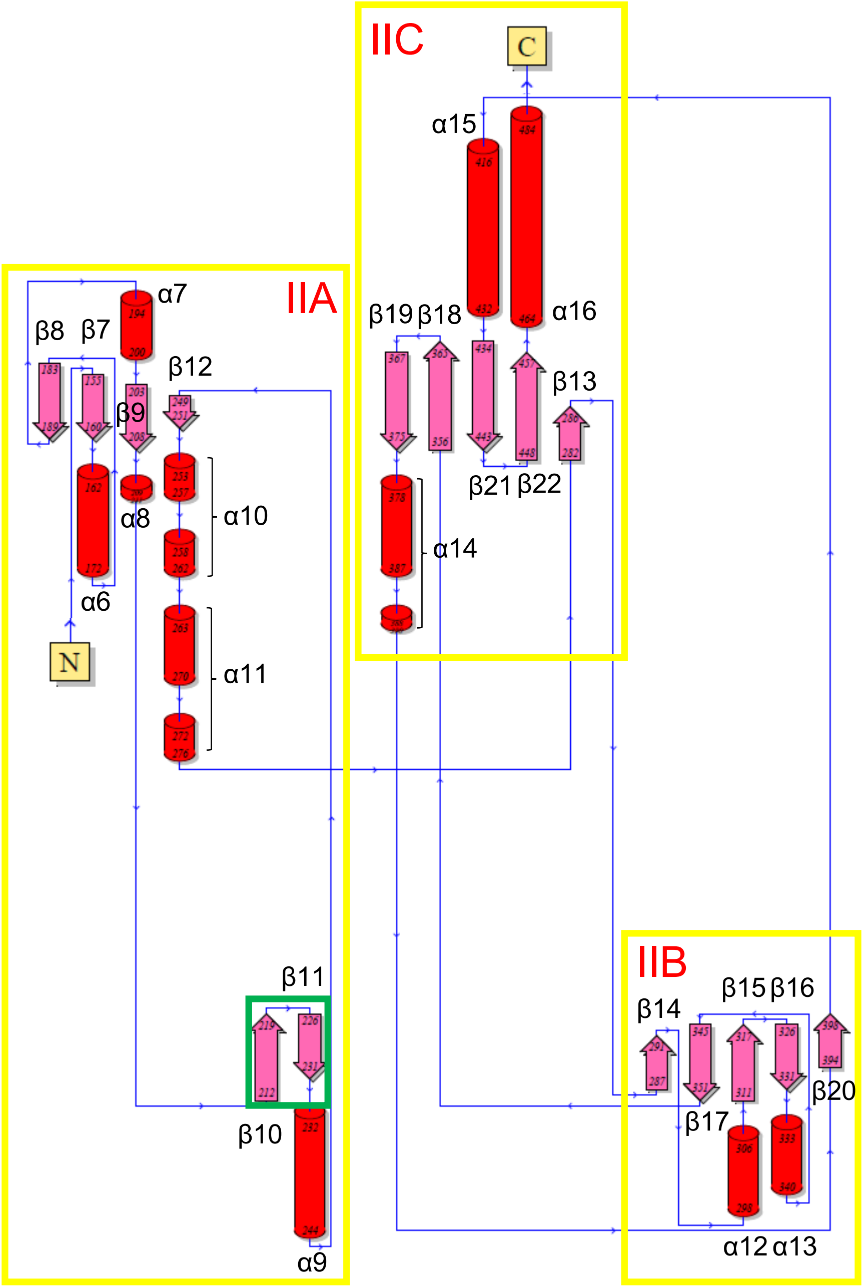

IIC
C
α15
α16
β19 β18
β13
β21 β22
α14
IIA
α7
β8 β7
β12
β9
α8
α10
α6
N
α11
β11
β10
α9
IIB
β15 β16
β14
β20
β17
α12 α13

Figure S7

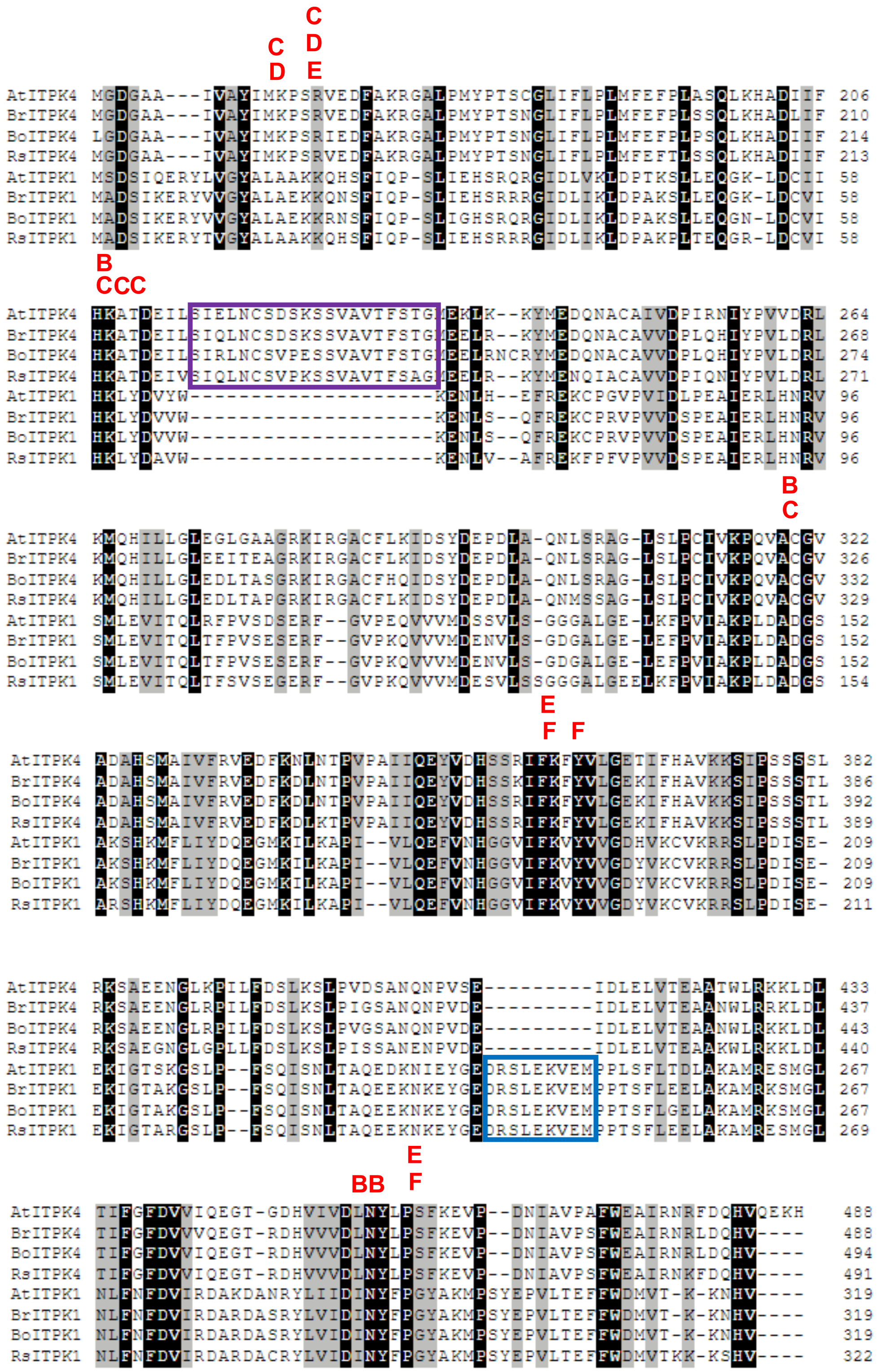
C
C D
D E
AtITPK4 MGDGAA---IVAYIMKPSRVEDFAKRGALPMYPTSCGLIFLPLMFEFPLASQLKHADIIF 206
BrITPK4 MGDGAA---IVAYIMKPSRVEDFAKRGALPMYPTSNGLIFLPLMFEFPLSSQLKHADLIF 210
BoITPK4 LGDGAA---IVAYIMKPSRIEDFAKRGALPMYPTSNGLIFLPLMFEFPLPSQLKHADIIF 214
RsITPK4 MGDGAA---IVAYIMKPSRVEDFAKRGALPMYPTSNGLIFLPLMFEFTLSSQLKHADIIF 213
AtITPK1 MSDSIQERYLVGYALAAKKQHSFIQP-SLIEHSRQRGIDLVKLDPTKSLLEQGK-LDCII 58
BrITPK1 MADSIKERYVVGYALAEKKQNSFIQP-SLIEHSRRRGIDLIKLDPAKSLLEQGK-LDCVI 58
BoITPK1 MADSIKERYVVGYALAEKKRNSFIQP-SLIGHSRQRGIDLIKLDPAKSLLEQGN-LDCVI 58
RsITPK1 MADSIKERYTVGYALAAKKQHSFIQP-SLIEHSRRRGIDLIKLDPAKPLTEQGR-LDCVI 58
B
CCC
AtITPK4 HKATDEILSIELNCSDSKSSVAVTFSTGMEKLK--KYMEDQNACAIVDPIRNIYPVVDRL 264
BrITPK4 HKATDEILSIQLNCSDSKSSVAVTFSTGMEELR--KYMEDQNACAVVDPLQHIYPVLDRL 268
BoITPK4 HKATDEILSIRLNCSVPESSVAVTFSTGMEELRNCRYMEDQNACAVVDPLQHIYPVLDRL 274
RsITPK4 HKATDEIVSIQLNCSVPKSSVAVTFSAGMEELR--KYMENQIACAVVDPIQNIYPVLDRL 271
AtITPK1 HKLYDVYW--------------------KENLH--EFREKCPGVPVIDLPEAIERLHNRV 96
BrITPK1 HKLYDVVW--------------------KENLS--QFREKCPRVPVVDSPEAIERLHNRV 96
BoITPK1 HKLYDVVW--------------------KENLS--QFREKCPRVPVVDSPEAIERLHNRV 96
RsITPK1 HKLYDAVW--------------------KENLV--AFREKFPFVPVVDSPEAIERLHNRV 96
B
C
AtITPK4 KMQHILLGLEGLGAAGRKIRGACFLKIDSYDEPDLA-QNLSRAG-LSLPCIVKPQVACGV 322
BrITPK4 KMQHILLGLEEITEAGRKIRGACFLKIDSYDEPDLA-QNLSRAG-LSLPCIVKPQVACGV 326
BoITPK4 KMQHILLGLEDLTASGRKIRGACFHQIDSYDEPDLA-QNLSRAG-LSLPCIVKPQVACGV 332
RsITPK4 KMQHILLGLEDLTAPGRKIRGACFLKIDSYDEPDLA-QNMSSAG-LSLPCIVKPQVACGV 329
AtITPK1 SMLEVITQLRFPVSDSERF--GVPEQVVVMDSSVLS-GGGALGE-LKFPVIAKPLDADGS 152
BrITPK1 SMLEVITQLTFPVSESERF--GVPKQVVVMDENVLS-GDGALGE-LEFPVIAKPLDADGS 152
BoITPK1 SMLEVITQLTFPVSESERF--GVPKQVVVMDENVLS-GDGALGE-LEFPVIAKPLDADGS 152
RsITPK1 SMLEVITQLTFSVSEGERF--GVPKQVVVMDESVLSSGGGALGEELKFPVIAKPLDADGS 154
E
F F
AtITPK4 ADAHSMAIVFRVEDFKNLNTPVPAIIQEYVDHSSRIFKFYVLGETIFHAVKKSIPSSSSL 382
BrITPK4 ADAHSMAIVFRVEDFKDLNTPVPAIIQEYVDHSSKIFKFYVLGEKIFHAVKKSIPSSSTL 386
BoITPK4 ADAHSMAIVFRVEDFKDLNTPVPAIIQEYVDHSSRIFKFYVLGEKIFHAVKKSIPSSSTL 392
RsITPK4 ADAHSMAIVFRVEDFKDLKTPVPAIIQEYVDHSSRIFKFYVLGEKIFHAVKKSIPSSSTL 389
AtITPK1 AKSHKMFLIYDQEGMKILKAPI--VLQEFVNHGGVIFKVYVVGDHVKCVKRRSLPDISE- 209
BrITPK1 AKSHKMFLIYDQEGMKILKAPI--VLQEFVNHGGVIFKVYVVGDYVKCVKRRSLPDISE- 209
BoITPK1 AKSHKMFLIYDQEGMKILKAPI--VLQEFVNHGGVIFKVYVVGDYVKCVKRRSLPDISE- 209
RsITPK1 ARSHKMFLIYDQEGMKILKAPI--VLQEFVNHGGVIFKVYVVGDYVKCVKRRSLPDISE- 211
AtITPK4 RKSAEENGLKPILFDSLKSLPVDSANQNPVSE---------IDLELVTEAATWLRKKLDL 433
BrITPK4 RKSAEENGLRPILFDSLKSLPIGSANQNPVDE---------IDLELVTEAANWLRRKLDL 437
BoITPK4 RKSAEENGLRPILFDSLKSLPVGSANQNPVDE---------IDLELVTEAANWLRKKLDL 443
RsITPK4 RKSAEGNGLGPLLFDSLKSLPISSANENPVDE---------IDLELVTEAAKWLRKKLDL 440
AtITPK1 EKIGTSKGSLP--FSQISNLTAQEDKNIEYGEDRSLEKVEMPPLSFLTDLAKAMRESMGL 267
BrITPK1 EKIGTAKGSLP--FSQISNLTAQEEKNKEYGEDRSLEKVEMPPTSFLEELAKAMRKSMGL 267
BoITPK1 EKIGTAKGSLP--FSQISNLTAQEEKNKEYGEDRSLEKVEMPPTSFLGELAKAMRKSMGL 267
RsITPK1 EKIGTARGSLP--FSQISNLTAQEEKNKEYGEDRSLEKVEMPPTSFLEELAKAMRESMGL 269
E
BB F
AtITPK4 TIFGFDVVIQEGT-GDHVIVDLNYLPSFKEVP--DNIAVPAFWEAIRNRFDQHVQEKH 488
BrITPK4 TIFGFDVVVQEGT-RDHVVVDLNYLPSFKEVP--DNIAVPSFWEAIRNRLDQHV---- 488
BoITPK4 TIFGFDVVIQEGT-RDHVVVDLNYLPSFKEVP--DNIAVPSFWEAIRNRLDQHV---- 494
RsITPK4 TIFGFDVVIQEGT-RDHVVVDLNYLPSFKEVP--DNIAVPSFWEAIRNKFDQHV---- 491
AtITPK1 NLFNFDVIRDAKDANRYLIIDINYFPGYAKMPSYEPVLTEFFWDMVT-K-KNHV---- 319
BrITPK1 NLFNFDVIRDARDASRYLVIDINYFPGYAKMPSYEPVLTEFFWDMVT-K-KNHV---- 319
BoITPK1 NLFNFDVIRDARDASRYLVIDINYFPGYAKMPSYEPVLTEFFWDMVT-K-KNHV---- 319
RsITPK1 NLFNFDVIRDARDACRYLVIDINYFPGYAKMPSYEPVLTEFFWDMVTKK-KSHV---- 322

Figure S8

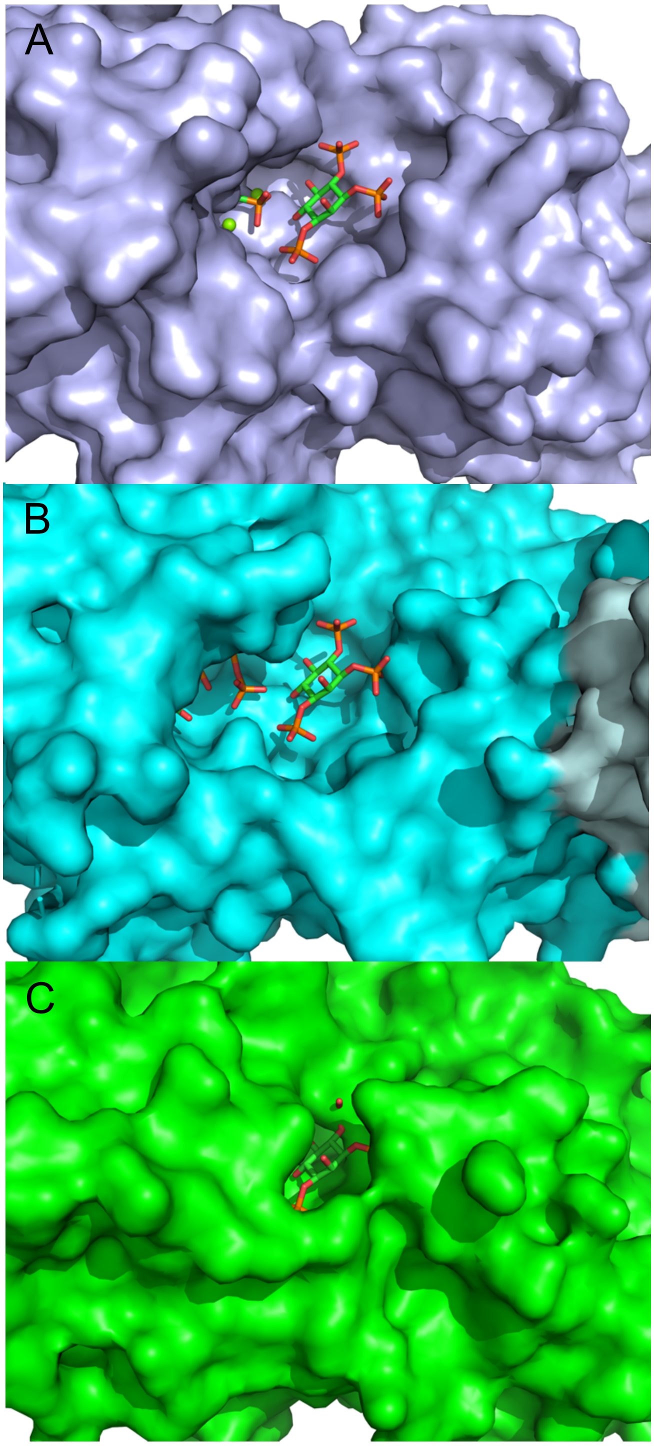

Figure S9

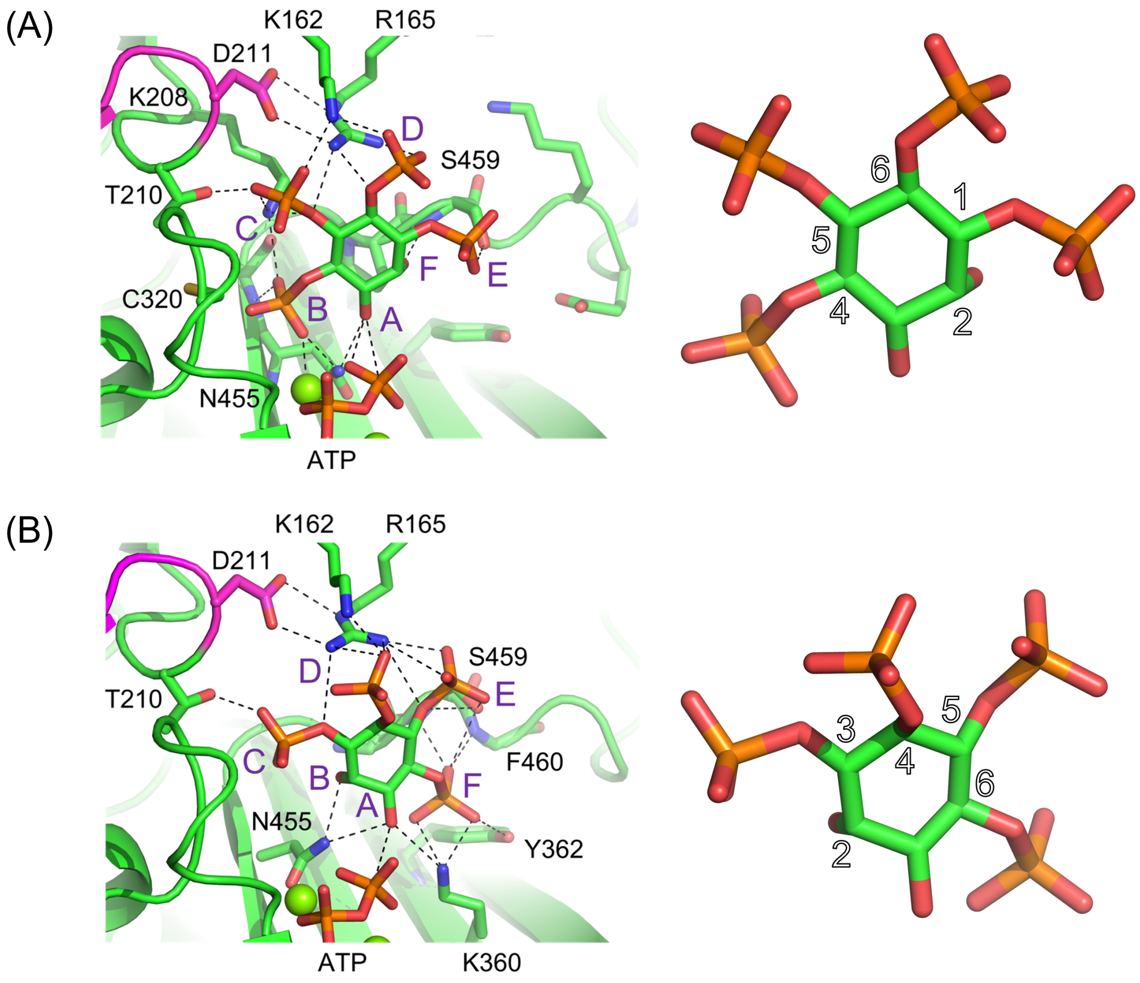
(A)
K162
R165
D211
K208
D
S459
T210
C
F
E
C320
B
A
N455
ATP
6
1
5
4
2
(B)
K162
R165
D211
S459
D
E
T210
F460
C
B
A
F
N455
Y362
ATP
K360
3
5
4
6
2

Figure S10

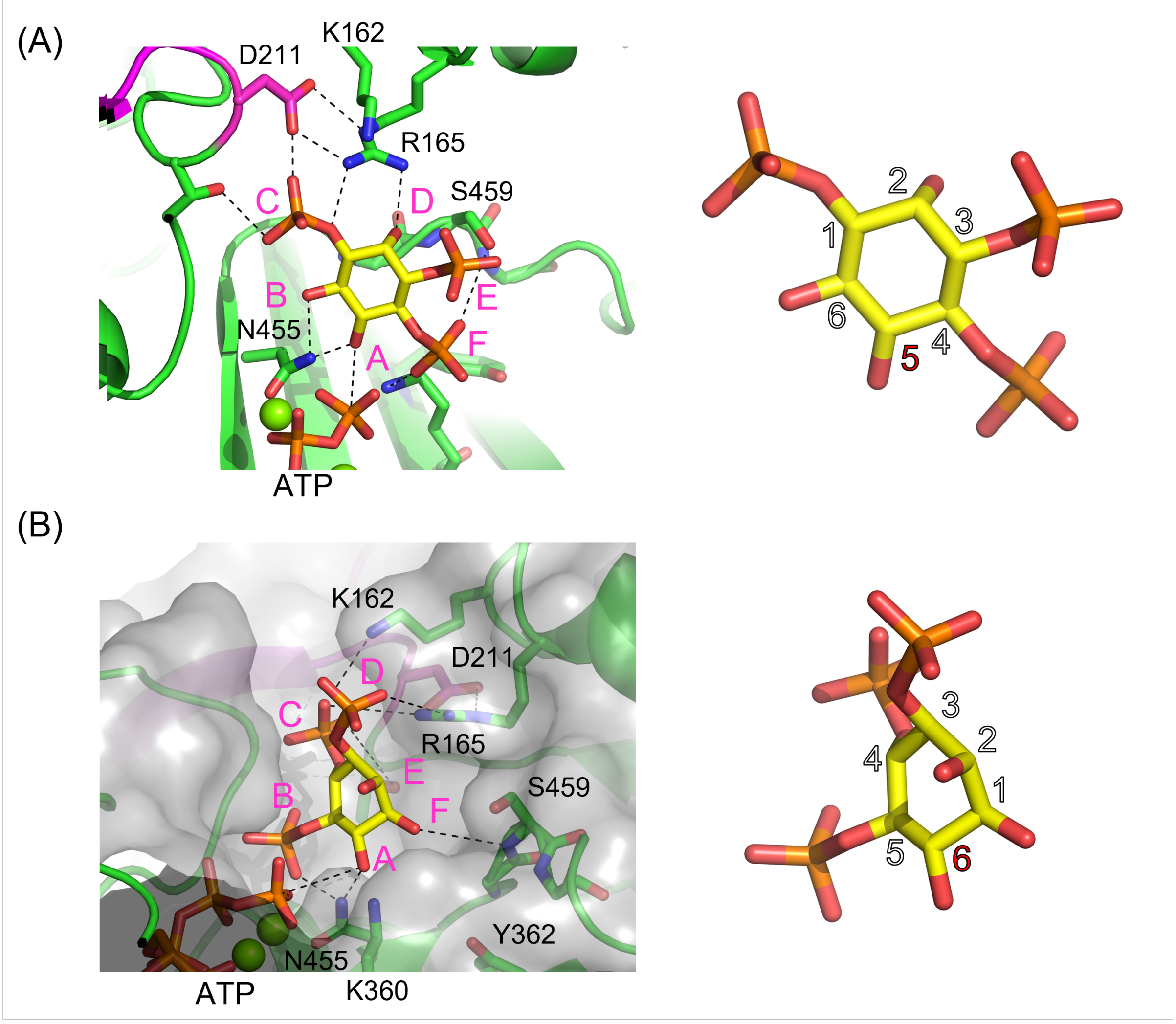
(A)
K162
D211
R165
S459
C
D
B
E
N455
F
A
ATP
1
2
3
4
5
6
(B)
K162
D211
D
C
R165
E
B
F
S459
A
Y362
N455
ATP
K360
3
2
4
1
5
6

Figure S11

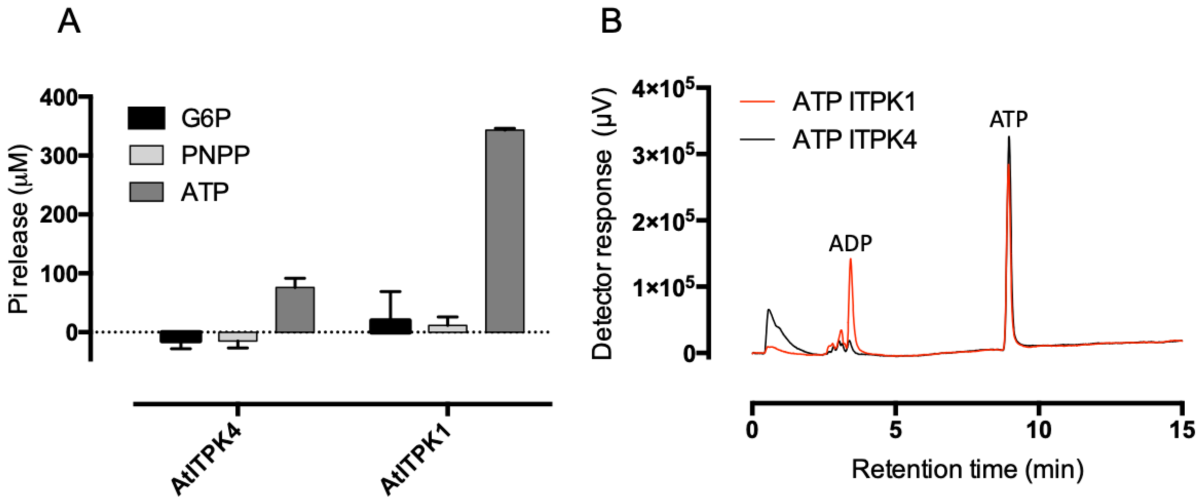

C

Enzyme	-	ITPK1	ITPK1	ITPK4	ITPK4
ATP	✓	✓	✓	✓	✓
InsP	-	-	Ins(3,4,5,6)P_4	-	Ins(1,4,6)P_3
% ADP	1	32	81	4	21
% InsP product	-	-	91	-	18

Figure S12

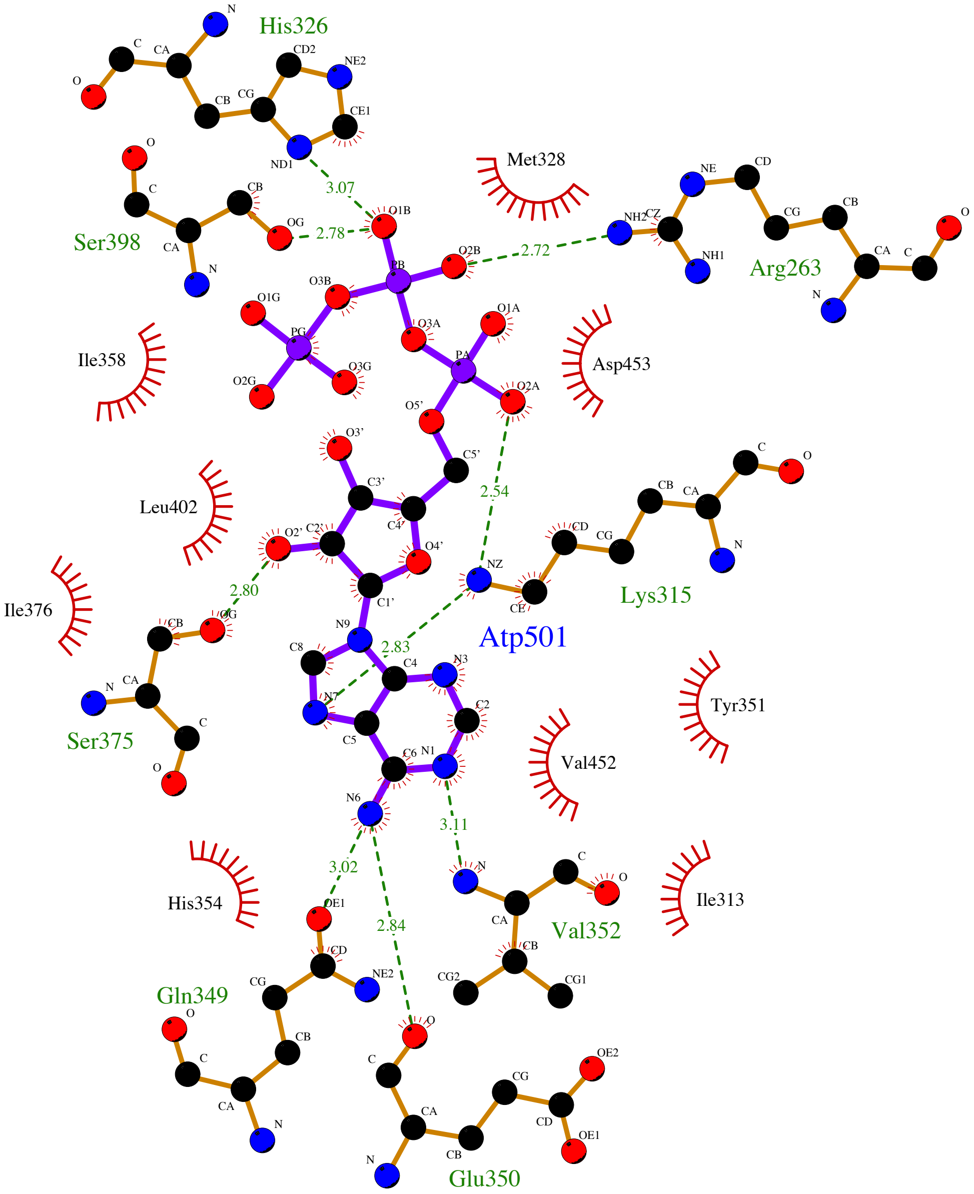
His326
Met328
Ser398
Arg263
Ile358
Asp453
Leu402
Ile376
Lys315
Atp501
Ser375
Tyr351
Val452
His354
Ile313
Val352
Gln349
Glu350
3.07
2.78
2.72
2.54
2.80
2.83
3.11
3.02
2.84

.

Figure S13

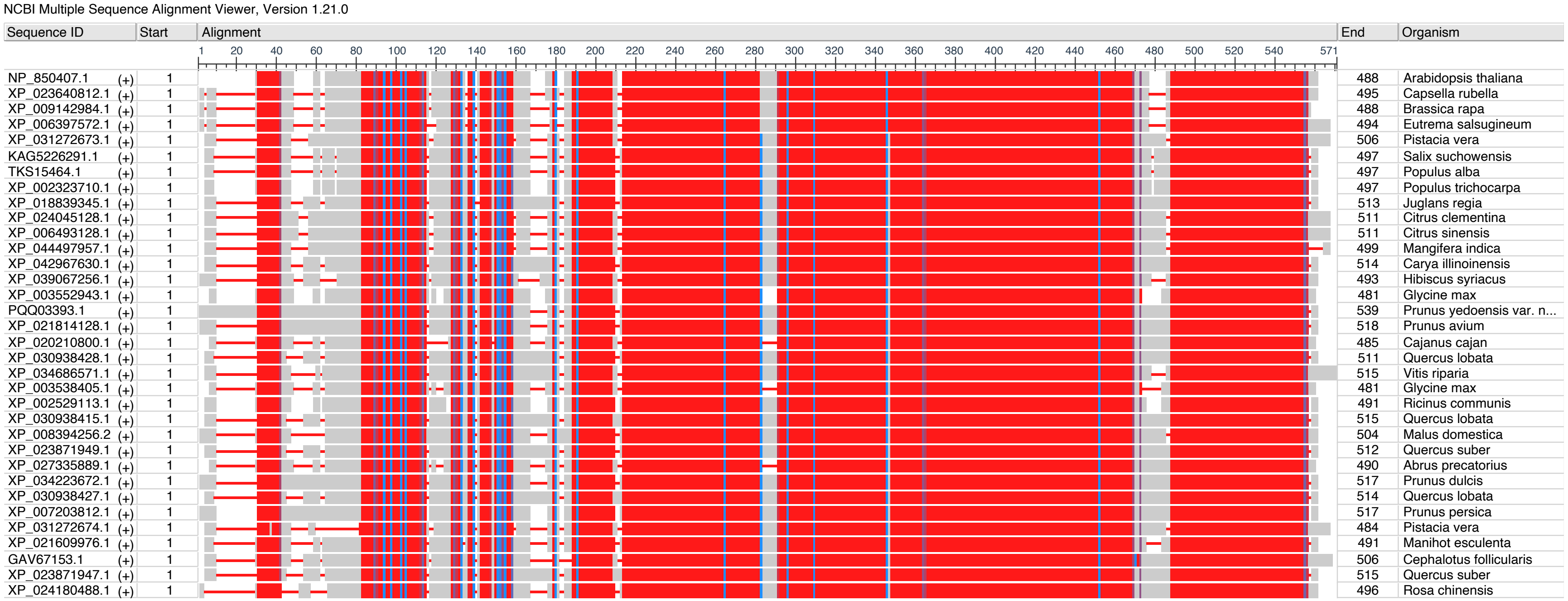
NCBI Multiple Sequence Alignment Viewer, Version 1.21.0
Sequence ID
Start
Alignment
End
Organism
1 20 40 60 80 100 120 140 160 180 200 220 240 260 280 300 320 340 360 380 400 420 440 460 480 500 520 540 571
NP_850407.1 (+) 1 488 Arabidopsis thaliana
XP_023640812.1 (+) 1 495 Capsella rubella
XP_009142984.1 (+) 1 488 Brassica rapa
XP_006397572.1 (+) 1 494 Eutrema salsugineum
XP_031272673.1 (+) 1 506 Pistacia vera
KAG5226291.1 (+) 1 497 Salix suchowensis
TKS15464.1 (+) 1 497 Populus alba
XP_002323710.1 (+) 1 497 Populus trichocarpa
XP_018839345.1 (+) 1 513 Juglans regia
XP_024045128.1 (+) 1 511 Citrus clementina
XP_006493128.1 (+) 1 511 Citrus sinensis
XP_044497957.1 (+) 1 499 Mangifera indica
XP_042967630.1 (+) 1 514 Carya illinoinensis
XP_039067256.1 (+) 1 493 Hibiscus syriacus
XP_003552943.1 (+) 1 481 Glycine max
PQQ03393.1 (+) 1 539 Prunus yedoensis var. n...
XP_021814128.1 (+) 1 518 Prunus avium
XP_020210800.1 (+) 1 485 Cajanus cajan
XP_030938428.1 (+) 1 511 Quercus lobata
XP_034686571.1 (+) 1 515 Vitis riparia
XP_003538405.1 (+) 1 481 Glycine max
XP_002529113.1 (+) 1 491 Ricinus communis
XP_030938415.1 (+) 1 515 Quercus lobata
XP_008394256.2 (+) 1 504 Malus domestica
XP_023871949.1 (+) 1 512 Quercus suber
XP_027335889.1 (+) 1 490 Abrus precatorius
XP_034223672.1 (+) 1 517 Prunus dulcis
XP_030938427.1 (+) 1 514 Quercus lobata
XP_007203812.1 (+) 1 517 Prunus persica
XP_031272674.1 (+) 1 484 Pistacia vera
XP_021609976.1 (+) 1 491 Manihot esculenta
GAV67153.1 (+) 1 506 Cephalotus follicularis
XP_023871947.1 (+) 1 515 Quercus suber
XP_024180488.1 (+) 1 496 Rosa chinensis

Figure S14

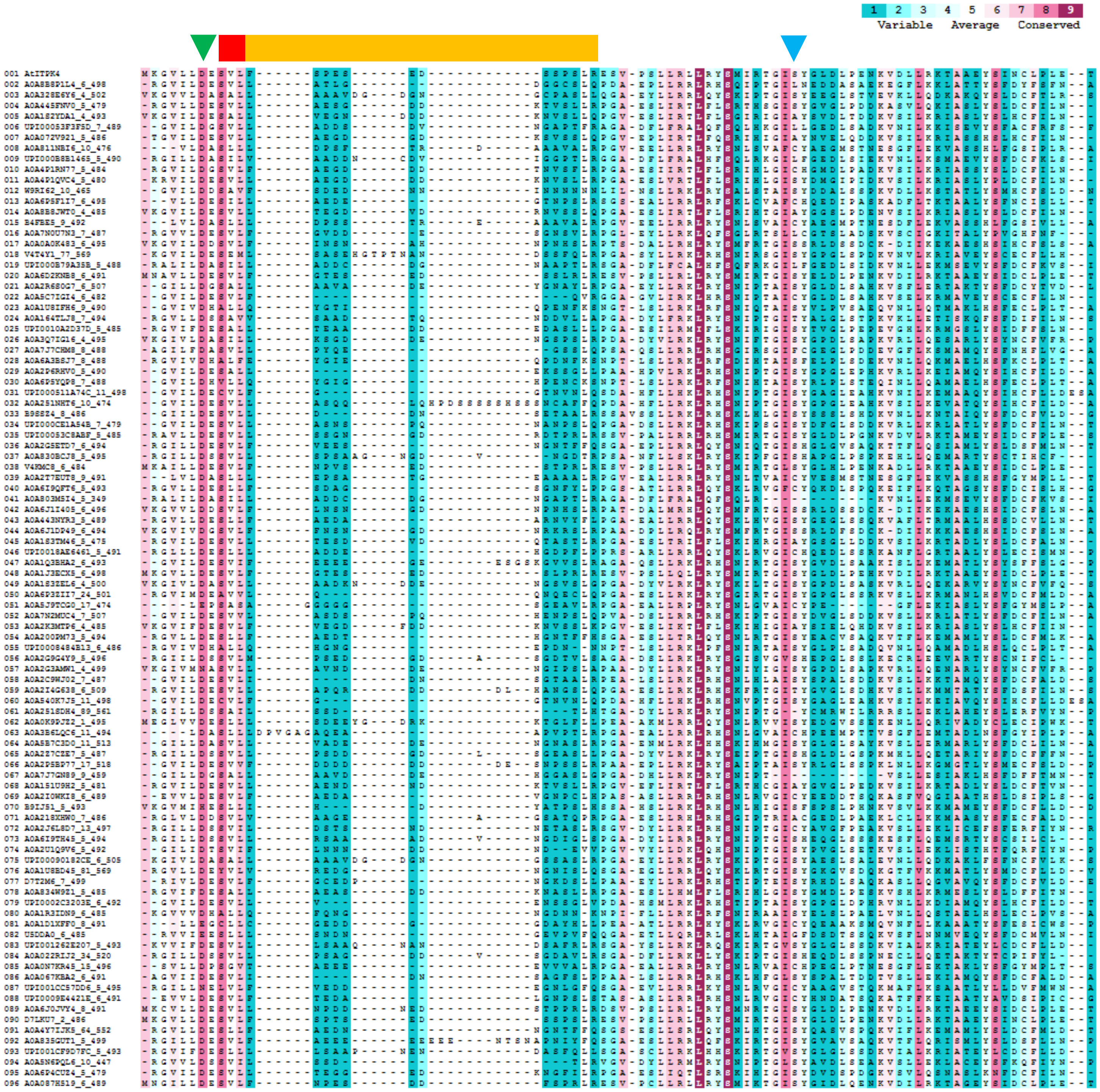

Figure S14 (continued)

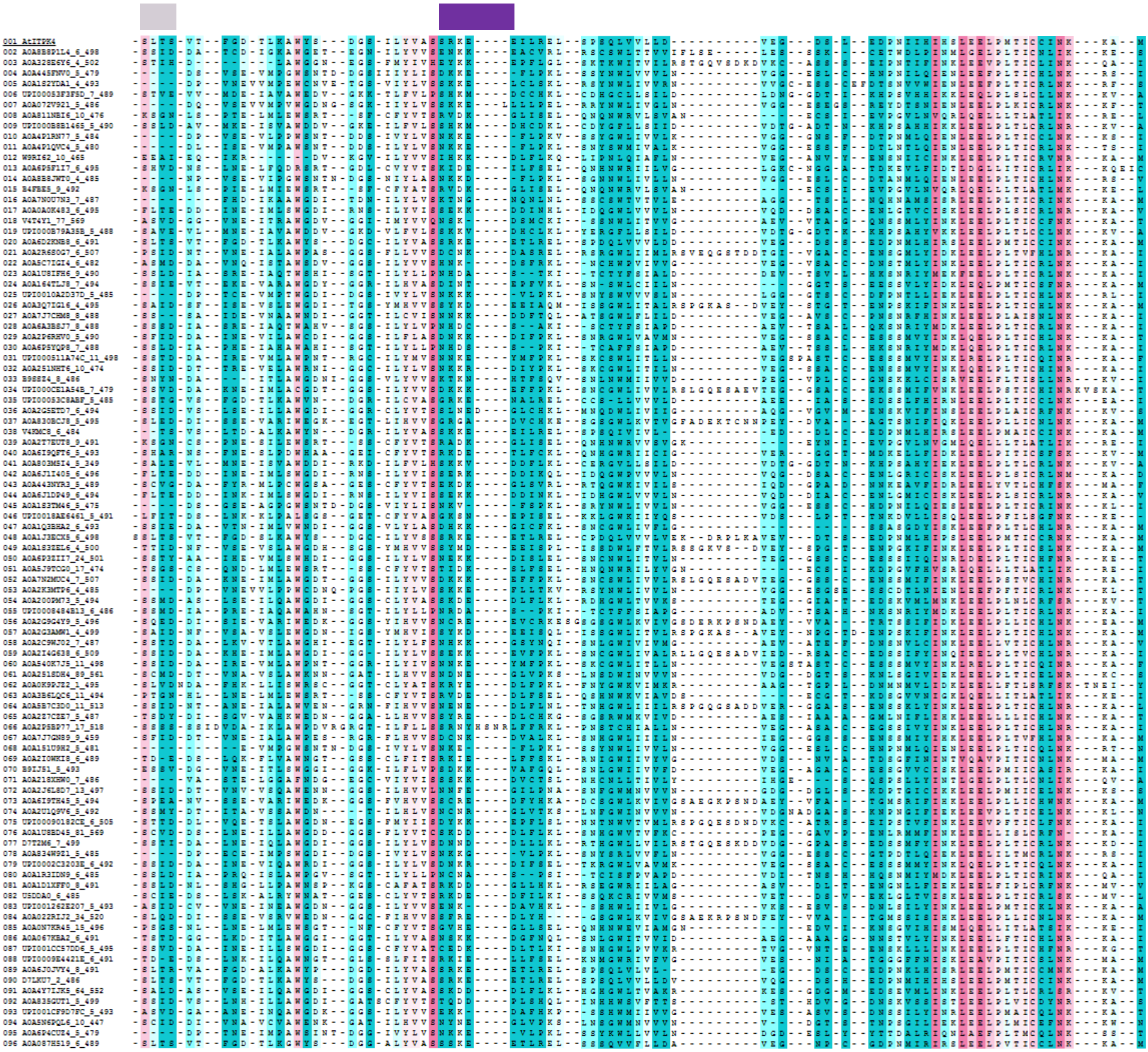

Table S1. Residues of *At*ITPK4 predicted to interact (≤4Å) with Ins(1,4,5,6)P_4 and Ins(3,4,5,6)P_4 in energy minimized models.

Specificity pocket	Ins(1,4,5,6)P_4		Ins(3,4,5,6)P_4	
**A**	3-OH	N455	1-OH	N455
**B**	4-PO_4	K208	2-OH	N455
		C320		
		N455		
		Y456		
**C**	5-PO_4	K162	3-PO_4	R165
		R165		K208
		K208		T210
		T210		D211
		D211		C320
		C320		
**D**	6-PO_4	K162	4-PO_4	K162
		R165		R165
**E**	1-PO_4	S459	5-PO_4	R165
				K360
				S459
**F**	2-OH	S459	6-PO_4	K360
				Y362
				S459
